# Mosaic and Concerted Evolution in the Visual System of Birds

**DOI:** 10.1371/journal.pone.0090102

**Published:** 2014-03-12

**Authors:** Cristián Gutiérrez-Ibáñez, Andrew N. Iwaniuk, Bret A. Moore, Esteban Fernández-Juricic, Jeremy R. Corfield, Justin M. Krilow, Jeffrey Kolominsky, Douglas R. Wylie

**Affiliations:** 1 Centre for Neuroscience, University of Alberta, Edmonton, Canada; 2 Department of Neuroscience, Canadian Centre for Behavioural Neuroscience, University of Lethbridge, Lethbridge, Canada; 3 Department of Biological Sciences, Purdue University, West Lafayette, Indiana, United States of America; 4 Department of Psychology, University of Alberta, Edmonton, Canada; Pennsylvania State University, United States of America

## Abstract

Two main models have been proposed to explain how the relative size of neural structures varies through evolution. In the mosaic evolution model, individual brain structures vary in size independently of each other, whereas in the concerted evolution model developmental constraints result in different parts of the brain varying in size in a coordinated manner. Several studies have shown variation of the relative size of individual nuclei in the vertebrate brain, but it is currently not known if nuclei belonging to the same functional pathway vary independently of each other or in a concerted manner. The visual system of birds offers an ideal opportunity to specifically test which of the two models apply to an entire sensory pathway. Here, we examine the relative size of 9 different visual nuclei across 98 species of birds. This includes data on interspecific variation in the cytoarchitecture and relative size of the isthmal nuclei, which has not been previously reported. We also use a combination of statistical analyses, phylogenetically corrected principal component analysis and evolutionary rates of change on the absolute and relative size of the nine nuclei, to test if visual nuclei evolved in a concerted or mosaic manner. Our results strongly indicate a combination of mosaic and concerted evolution (in the relative size of nine nuclei) within the avian visual system. Specifically, the relative size of the isthmal nuclei and parts of the tectofugal pathway covary across species in a concerted fashion, whereas the relative volume of the other visual nuclei measured vary independently of one another, such as that predicted by the mosaic model. Our results suggest the covariation of different neural structures depends not only on the functional connectivity of each nucleus, but also on the diversity of afferents and efferents of each nucleus.

## Introduction

In recent years, there has been an increased interest in understanding the principles and processes that govern brain evolution [1]. A major goal has been to understand how differences in the absolute and relative size of different neural structures evolve and two models have been proposed. In the concerted evolution model, developmental constraints cause different parts of the brain to vary in size in a coordinated manner [2], [3]. Thus, if there is selective pressure to increase the size of a specific brain region, the rest of the brain will increase in size as well. In the mosaic evolution model, there are no such constraints and individual brain structures can vary in size independently of each other [4]–[6]. Most studies to date have tested these models at an anatomically crude level, comparing variation of the relative size of large subdivision of the brain, such as telencephalon, thalamus, cerebellum and brainstem (see [7] for an exception]). The results of these analyses support either model of evolutionary change depending upon which clade is being examined (e.g. [4]–[6], [8]).

A possible drawback of the use of major subdivisions of the brain is that they do not represent functional units; each region contains multiple independent motor and sensory pathways. This means that the size of these different regions of the brain is the result of a complex combination of multiple selection pressures and constraints affecting several motor and sensory pathways. Selective hypertrophy of neural structures related to sensory (e.g. [9]–[12]), and motor (e.g. [13], [14]) specializations are well documented, but the majority of these studies are restricted to one structure and therefore it is unclear if functionally and anatomically related nuclei evolve according to a concerted or mosaic model of evolutionary change. While some recent studies have suggested concerted evolution in some sensory pathways of birds (e.g. [15]–[17]), no study has specifically set out to test these two models at the level of specific neural pathways.

**Figure 1:**
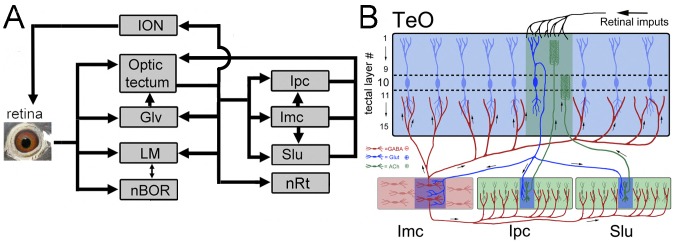
Connectivity of the avian visual system and the isthmo-tectal circuit. **A**, illustrates some of the connectivity in the visual pathways in birds. The black arrows show the projections from one structure to the other. The optic tectum (TeO), the nucleus of the basal optic root (nBOR), the nucleus lentiformis mesenscephali (LM) and the ventral geniculate nucleus (GLv) all receive projections from the contralateral retina. The isthmo-optic nucleus (ION), which projects to the retina, GLv, LM, the nucleus rotundus (nRt), the magnocellular and parvocellular portions of nucleus isthmi (Imc, Ipc) and the nucleus semilunaris (SLu) all receive projections from TeO. Several of the nuclei are also interconnected, like LM and nBOR or Imc, Ipc, and SLu. **B**, illustrates in detail the isthmo-tectal circuit. Imc, Ipc and SLu receive a topographic, excitatory projection from cells in layer 10 of the TeO (blue cells). Ipc and SLu send back excitatory projections to TeO in a topographic manner (green cells). Imc neurons on the other hand are GABAergic [50], [51] and send an ‘antitopographic’ projection to Ipc, SLu or to the deep layers of TeO [52].

The visual system of birds is a good candidate to study the covariation of the relative size of nuclei that belong to the same pathway or sensory modalities. In birds, like in all vertebrates, projections from the retina go to several retinorecipient nuclei, which give rise to several parallel visual pathways. The main retinorecipient structure is the optic tectum (TeO), a multilayered structure that in pigeons receives more than 90% of retinal projections and forms part of the tectofugal pathway (Fig. 1A; [18]–[20]). The tectofugal pathway is also comprised of the nucleus rotundus (nRt) in the thalamus and the entopallium (E) in the telencephalon. This pathway is involved in processing brightness, colour, pattern discrimination, simple motion and looming stimuli [21]–[25]. A second pathway is the thalamofugal pathway, which includes the lateral part of the nucleus dorsolateralis anterios thalami (DLL) in the dorsal thalamus and the Wulst (also known as the hyperpallium [26], [27]). Other retinorecipient nuclei in birds include the nucleus lentiformis mesencephali (LM) and the nucleus of the basal optic root (nBOR; [28]–[31]) both of which are involved in the generation of the optokinetic response [32], and the ventral lateral geniculate nucleus (GLv), whose function remains largely unclear (see [33]–[37] for some proposed functions). Besides all receiving retinal projections, these nuclei are all interconnected with one another. For example, GLv and LM receive projections from TeO [38]–[41] and LM and nBOR have massive reciprocal projections [42]. The isthmo optic nucleus (ION), a small nucleus in the isthmal region, receives projections from the tectum and sends projections to the retina, thus creating a loop between retina, TeO and ION (reviewed in [43]). Another group of nuclei interconnected with TeO is the isthmal complex, which is composed of the magnocellular and parvocellular parts of the nucleus isthmi (Imc and Ipc) and the nucleus semilunaris (SLu). Each of these nuclei receives a prominent, retinotopically organized visual projection from the ipsilateral TeO, specifically from ‘shepherd's crook’ neurons [38], [44]–[47]. Ipc and SLu neurons are cholinergic (Fig. 1B; [48], [49]) and project back to TeO in a precise homotopic fashion (Fig. 1B; [20], [38], [44], [46], [47]). Imc neurons are GABAergic (Fig. 1B; [50], [51]) and send an anti-topographic projection to Ipc, SLu or to the deep layers of TeO (Fig. 1B; [52]). By anti-topographic, we mean that Imc neurons project broadly to the TeO, Ipc and SLu, except to the locus from which they receive projections (Fig. 1B).

**Figure 2:**
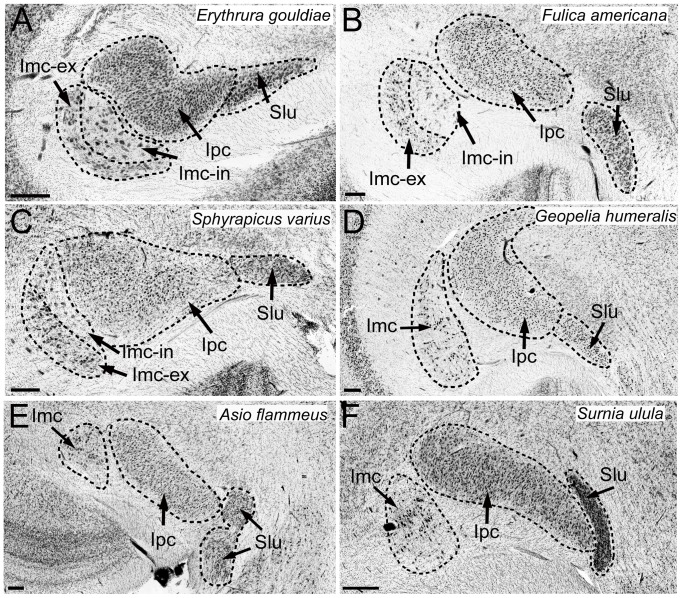
Location, borders and cytoarchitecture of the isthmal complex. Photomicrographs showing the location and borders of the three isthmal nuclei, the magnocellular and parvocellular portions of nucleus isthmi (**Imc**, **Ipc**) and the nucleus semilunaris (**SLu**) in four species of birds. **A**–**C** show the isthmal complex in the three different groups of birds that exhibited a Imc segregated in two layers, the internal subdivision of the Imc (Imc-in) and the external subdivision of the Imc (Imc-ex). **A** shows a songbird (Passeriformes), the Gouldian Finch (*Erythrura gouldiae*). **B** shows a Gruiform, the American Coot (*Fulica Americana*). **C** shows a woodpecker (Piciformes), the Yellow-bellied Sapsucker (*Sphyrapicus varius*); **D** shows a pigeon (Columbiformes), the Bar-shouldered Dove (*Geopelia humeralis*). **E** and **F** show two species of owls (Strigiformes), the Short-eared Owl (*Asio flammeus*) and the Northern Hawk Owl (*Surnia ulula*).

Several comparative studies have shown great variation in the relative size of visual nuclei in birds, both among and within orders [12], [17], [53], [54]. For example, Iwaniuk and Wylie [12] showed that LM, but not GLv, nBOR or TeO, is greatly enlarged in hummingbirds. Similar volumetric studies have shown a reduction in size of the TeO and the rest of the tectofugal pathway in in owls, parrots and waterfowls compared to other birds [15] and great variation in the relative size of the ION among and within orders [55]. The heavily interconnected circuitry (Fig. 1) and known variation in the relative size of some of the nuclei therefore makes the visual system ideal for testing whether the mosaic or concerted models of brain evolution applies to an entire sensory pathway. Here, we examine the relative size of 9 different visual nuclei in 98 species of birds belonging to 16 different orders. This includes data on interspecific variation in the cytoarchitecture and relative size of the isthmal nuclei (Ipc, Imc, SLu), which has not been previously reported. Specifically, we tested for interspecific differences in Imc related to cytoarchitectural differences. In the chick (*Gallus domesticus*), Imc is composed of two different cells types; one cell type projects to Ipc and SLu, and the other cells project to TeO (Fig. 1B; [47], [52]). Recently, Faunes et al. [56] showed that in the zebra finch (*Taeniopygia guttata*), these two cells types are segregated in two subdivisions, which are identified as the external (Imc-ex) and internal (Imc-in) Imc (e.g. Fig. 2A–C). Further, this segregation is likely present in all songbirds (Passeriformes), but not in most other birds with the exception of coots (Gruiformes) and woodpeckers and allies (Piciformes) (Fig. 2B–C). In vertebrates, lamination has evolved in several neural structures (for a review see [1]), which is likely related to an increase in the size of the structure and/or a need to minimize connection lengths and thereby increase processing power [1]. Recently, we have shown that in the ION, the presence of a clearly segregated cell layer and neuropil is related to an increase in the relative size of this nucleus [55]. Thus, it is possible that groups that have a segregated Imc have a relatively larger Imc than birds with a non-segregated Imc.

In addition to the descriptions and measurements of the isthmal nuclei, we used a combination of statistical analyses to test if visual nuclei evolve in a concerted or mosaic manner: i) phylogenetically corrected principal component analysis and, ii) evolutionary rates of change, on the absolute and relative size of the nine visual nuclei. Previous studies [4], [57] suggested that covariation in the size of different neural structures is related to their functional connectivity to one another. We therefore expected heavily interconnected and functionally related nuclei, such as the isthmal nuclei or LM and nBOR, to vary in relative size in a more concerted manner with each other than with other nuclei.

## Materials and Methods

### Ethics Statement

In all cases, the specimens were provided to us dead. Some of these species were collected dead from window strikes and culling operations in Australia by ANI under collection permits issued by the Victorian Department of Natural Resources and Environment. Other species were provided by other researchers, all of which had the correspondent capture/handling permits and/or ethics approval from their respective institutions. This includes Dr. Catherine Carr which had approval from the University of Maryland institutional animal care and use committee (IACUC), Dr. Lainy Day which had approval from the University of Mississippi IACUC, Dr. Ken Welch Jr. which had approval from the University of California, Riverside IACUC, and Dr. Tim R. Birkhead, who obtained specimens from local hunters in West Woodyates, Dorset, United Kingdom. Other specimens were provided by the Healesville Sanctuary (Healesville, Australia), the Springvale Veterinary Clinic (Springvale, Australia), the Melbourne Zoo (Melbourne, Australia) and the Alberta Institute for Wildlife Conservation (Madden, Canada) staff. In all of these cases, the specimens died from causes unrelated to this project.

Some of the songbird specimens in this study were captured in Tippecanoe County, Indiana, USA using mist-nets and live traps by BAM and EF-J. Authorization to capture these birds was obtained from the Indiana Department of Natural Resource and the U.S. Fish and Wildlife Service. Capture and study of all animals did not involve endangered or protected species. The Purdue Animal Care and Use Committee (protocol #1201000567) approved all capturing, handling, and experimental procedures with the birds (see table S1). Birds were housed indoors in cages (0.9 m×0.7 m×0.6 m) with 1–3 other individuals of the same species prior to tissue collection. They were kept on a 14∶10 hour light∶dark cycle and an ambient temperature of approximately 23°C. Food (millet, sunflower seeds and thistle seeds) and water was always provided *ad libitum*, and supplemented with mealworms (*Tenebrio molitor*) daily. Tissue collection began by euthanizing birds with CO_2_, followed by immediate removal of the eyes for a different study and the head (preserved in 4% paraformaldehyde) for this study.

### Measurements

We measured the relative volume of Ipc, Imc, SLu, ION, LM, GLv, nBOR, nRt and TeO in 100 specimens representing 98 species (table S1). Some of the values reported in this study, including the volume for ION in 81 of the species and volume for LM, nBOR, GLv, nRt and TeO in some of the species have been reported in previous work [12], [15], [17], [55]. For all specimens, the head was immersion-fixed in 4% paraformaldehyde in 0.1 M phosphate buffer. The brain was then extracted, weighed to the nearest milligram, cryoprotected in 30% sucrose in phosphate buffer, embedded in gelatin and sectioned in the coronal or sagittal plane on a freezing stage microtome at a thickness of 40 µm. Sections were collected in 0.1 M phosphate buffered saline, mounted onto gelatinized slides, stained with thionin and coverslipped with Permount (Fisher Scientific, Fair Lawn, New Jersey, USA). The olfactory bulbs were intact in all of the specimens that we collected and sectioned. All brains were cut following bird brain atlases [58], [59] in which the brainstem ends at the same rostrocaudal point as the cerebellum. In this manner, brain measurements were consistent among our specimens. Photomicrographs of every second or every fourth section were taken throughout the rostrocaudal extent of each nucleus using a Retiga EXi *FAST* Cooled mono 12-bit camera (Qimaging, Burnaby, BC, Canada) and OPENLAB Imaging system (Improvision, Lexington, MA, USA) attached to a compound light microscope (Leica DMRE, Richmond Hill, ON, Canada). For some brains, images of full sections were obtained with a digital slide scanner (Leica SCN400, Richmond Hill, ON, Canada) with a 20× objective.

Measurements of all the nuclei were taken directly from these photos with ImageJ (NIH, Bethesda, MD, USA; http://rsb.info.nih.gov/ij/) and volumes were calculated by multiplying the area in each section by the thickness of the section (40 µm) and the sampling interval. For those species represented by more than one specimen (table S1), the average of the measurements was taken as the species' given value.

### Borders of nuclei

**Figure 3:**
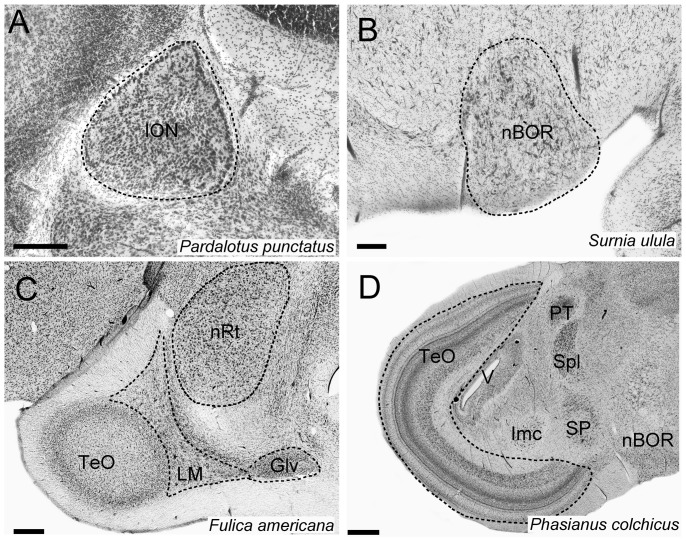
Location, borders and cytoarchitecture of other visual nuclei. Photomicrographs of coronal sections showing the location and borders of the different visual nuclei in birds. **A**, shows the isthmo optic nucleus (**ION**) in a songbird (Passeriformes) the Spotted Pardalote (*Pardalotus punctatus*). **B** shows the nucleus of the basal optic root (**nBOR**) in an owl (Strigiformes), the Northern Hawk Owl (*Surnia ulula*). **C** shows the nucleus lentiformis mesencephali (**LM**), the ventral part of the geniculate nucleus (**GLv**) and the nucleus rotundus (nRt) in a Gruiform, the American Coot (*Fulica americana*). **D** shows the optic tectum (**TeO**) in a gallinaceous bird (Galliformes) the Ring-necked Pheasant (*Phasianus colchicus*).

In all birds, Imc, Ipc and SLu were readily identifiable in Nissl stained sections. Imc and Ipc lie ventral and lateral to the ventricle and they are surrounded by fibers coming from the TeO. Ipc is medial and dorsal to Imc and is characterized by small, densely packed cells. In contrast, Imc is characterized by larger and more loosely arranged cells (Fig. 2). SLu is similar to Ipc, with small, darkly stained cells. It is ventral and medial to the posterior ventral tip of Ipc (Fig. 2) and lateral to the ventrolateral lemniscal nuclei. For the rest of the nuclei measured, we followed the same borders described in previous studies (Fig. 3: [12], [15], [55]).

### Material quality

**Figure 4:**
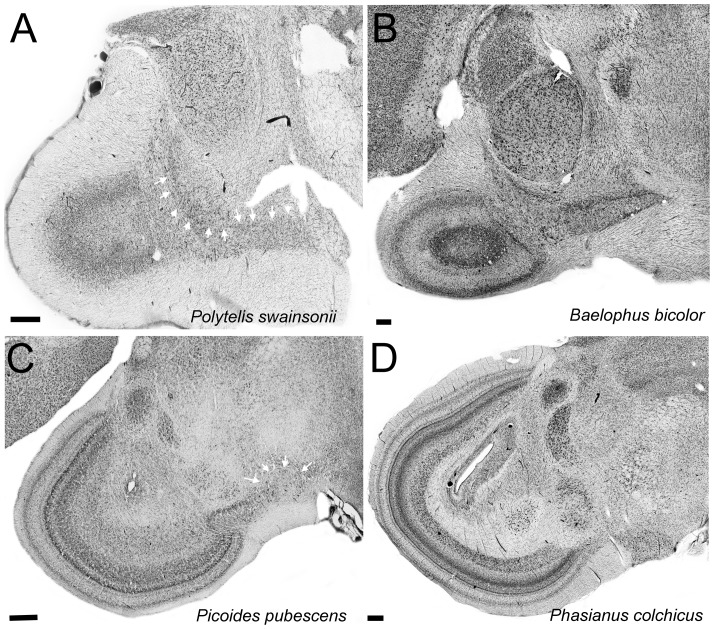
Tissue quality examples. Photomicrographs of Nissl stained coronal sections in four of the specimens used in this study. **A** and **C** show two of the lowest quality staining used in this study while **B** and **D** show sections equivalent to the ones showed in A and C in specimens with good quality of staining, Notice that even in **A** and **C**, the borders of visual structures measured in this study, like the nucleus lentiformis mecencephali (**LM**), the ventral part of the geniculate nucleus (**GLv**), the nucleus rotundus (**nRt**), the nucleus of the basal optic root (**nBOR**) and the optic tectum (**TeO**), are all clearly discernible. In **A**, the white arrows show the borders between LM and the nucleus laminaris precommissuralis (**LPC**) and also the dorsal border of GLv. In **C**, the white arrows show the border of nBOR. Scales bars = 400 µm.

As mentioned above, the material used in this study comes from a variety of sources and the brains were immersion fixed. This inevitably results in variation in the quality of the tissue because of variable fixation across specimens. Nonetheless, in this study we only used material where the borders of all the structures where clearly discernible. Figure 4 show a side-by-side comparison of some of the lowest (Fig. 4 A, C) and highest (Fig. 4 B, D) quality available. As it is clear from the photomicrograph, the borders of different visual nuclei like Glv, LM, nBOR and, TeO in the lower quality tissue are clearly discernible. Further, tissue in this condition only represents a small portion of the specimens used and the great majority (>80%) are in far better condition.

### Statistical analyses

To examine scaling relationships, we plotted the log_10_-transformed volume of each brain region against the log_10_-transformed brain volume minus the volume of each specific region [60]. Because of the close anatomical and functional relationship of the isthmal nuclei with the TeO (see introduction), we also examined the scaling relationships of these nuclei against the TeO.

Allometric equations were calculated with least squares linear regressions using: (1) species as independent data points, and (2) phylogenetic generalized least squares (PGLS) to account for phylogenetic relatedness [61], [62]. We applied two models of evolutionary change as implemented in the MATLAB program Regressionv2.m (available from T. Garland, Jr. on request; [63], [64]): Brownian motion (phylogenetic generalized least-squares or PGLS) and Ornstein–Uhlenbeck (OU) [64], [65]. Because different phylogenetic trees can yield different results [66] we tested two models based on the trees provided in Livezey and Zusi (2007; [67]), and Hackett et al. (2008; [68]). Resolution within each order was provided by order- and family-specific studies [69]–[78]. Phylogenetic trees, character matrices and phylogenetic variance-covariance matrices were constructed using Mequite/PDAP∶PDTREE software [79], [80] and the PDAP software package (available from T. Garland, Jr., upon request). Because the phylogeny was constructed from multiple sources, branch lengths were all set at 1, which provided adequately standardized branch lengths when checked using the procedures outlined in Garland et al. [81]. Unresolved nodes were treated as soft polytomies, with branch lengths between internal nodes set to zero [82]. Allometric equations based on standard statistics, and the PGLS and OU models, for each of the two trees, were calculated for: (1) visual nuclei volume against brain volume; and (2) Ipc, Imc and SLu volume against TeO volume. We also ran regression models that included order and the presence of one or two layers in Imc [56] as covariates of the volume of Ipc, Imc and SLu relative to both brain and TeO volume. Currently, there is no phylogenetically corrected pair wise comparison available and therefore Tukey HSD post hoc tests were only performed on non-phylogenetically corrected statistics.

Non-phylogenetically corrected statistics and post-hoc tests were performed using the software JMP (JMP, Version 10. SAS Institute Inc., Cary, NC, 1989–2007). Additionally, we calculated phylogeny-corrected 95% prediction intervals [60] using the PDAP module [79] of the Mesquite modular software package [80] to look for any significant outliers.

### Phylogenetic multivariate allometry analyses

To compare patterns of evolution among the different nuclei, we used maximum likelihood values for the lambda (λ) and alpha (α) parameters [83]. These parameters test for departure from a Brownian motion model of evolution where trait divergence accumulates in time in a stochastic manner. In the λ parameter test, a λ equal to 1 means a null Brownian motion model [83]. The α model is based on an OU process and estimates the strength of selection acting on the trait; the higher the value of α, the stronger the selective regime. As α becomes small the OU model is eventually reduced to a Brownian process. As α tends towards 1, the process will reduce to a model with one selective optimum but with no accelerated accumulation of divergence [84], [85]. P-values were obtained by comparing the models with the λ and α parameters to a null model of unconstrained Brownian motion with the log-likelihood statistic. The GEIGER [86] package in R [87] was used to estimate the values.

To test how the relative size of the nuclei vary with respect to each other, we used a correlation based principal components approach taking into account the phylogenetic relationships among species, using the *phyl.pca* functions of the PHYTOOLS package [88] in R. A multivariate allometric analysis has advantages over other methods, such as multiple regressions, in that it avoids problems with the adequate control of size when analyzing inter-correlation between structures, as well as problems of multicolinearity, which can arise because structure volumes are usually highly correlated with one another [89]–[91]. In any principal component analysis (PCA), where all variables are correlated with a size variable (in this case brain size), the first principal component corresponds to an isometric size variable [92]. In this sense, all other principal components will correspond to variance in the size of the different structures independent of brain size. The ratio between the loadings of any pair of variables in the first principal component (PC1) corresponds to the bivariate allometric coefficient of those variables [92]. Bivariate allometric coefficients close to 1 indicate isometry between two nuclei (i.e. both nuclei vary equally in size with changes in absolute size). Bivariate allometric coefficients that depart from 1 indicate positive or negative allometry between a pair of nuclei indicating that one nucleus changes in size disproportionally with respect to the other with changes in absolute size. Therefore, isometry between nuclei can be interpreted as indicative of concerted evolution between those nuclei while departure from it is an indication of mosaic evolution. In addition to running multivariate analysis on the absolute volume of the visual nuclei, we also performed a phylogenetically corrected PCA of the relative size of the nuclei. For this analysis, we used residuals from a phylogenetically-corrected least squares regression analysis, using the PHYTOOLS package in R. The residuals were then analyzed in the same fashion as the absolute volumes, using the *phyl.pca* functions of the PHYTOOLS package which performs a PCA that takes into account the phylogenetic relationships among species. As with the previous analyses, we used two different phylogenies [67], [68]. Because variation of the relative size of some of the nuclei departs from a Brownian motion evolutionary model (see results) we assumed both a Brownian motion and Pagel's λ [83] evolutionary model when performing the PCA analysis with the residuals.

All multivariate analyses included 94 of the 98 species because four species did not have a recognizable ION (see table S1, [55]) and the R function used to calculate the different parameters could not handle missing values.

## Results

### Isthmal nuclei cytoarchitecture

The cytoarchitectonics of the Ipc is similar across all birds that we examined (Fig. 2). The same is true for Imc with the exception of Passeriformes (songbirds), Gruiformes (coots and allies) and Piciformes (woodpeckers and allies) in which Imc cells are organized in two distinct layers as reported by Faunes et al. (2013; [56]) (Fig. 2A–C). We examined the cytoarchitectonical organization of Imc in 14 additional species of birds (13 songbirds and one Piciform) to the ones reported by Faunes et al. [56], all of which had two distinct layers of cells (table S1). We also found that owls have a distinct cytoarchitectonical organization of SLu. In 8 out of the 9 owl species in this study (the exception being the Northern Hawk Owl, *Surnia ulula*), SLu is divided into dorsal and ventral portions that are separated by a bundle of fibers that courses dorsal to Ipc, but ventral to the lateral part of the mesencephalic reticular formation, towards the brachium conjunctivum (Fig. 2E–F).

### Isthmal nuclei relative size

**Figure 5:**
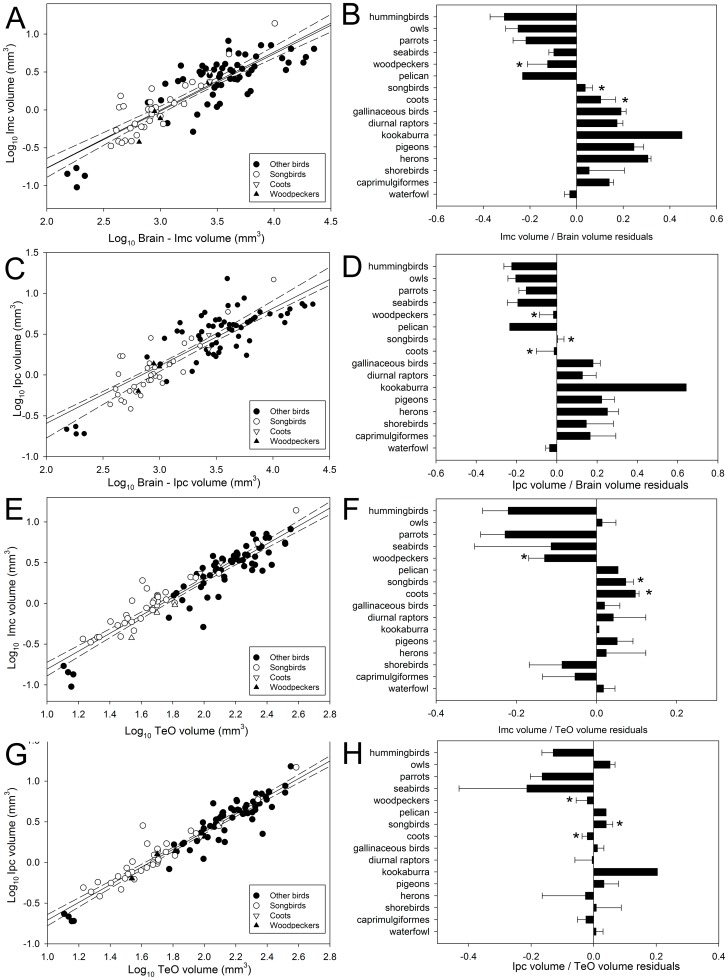
Relative size of the magnocellular and parvocellular portions of nucleus isthmi. Scatterplot of log-transformed volume of the magnocellular and parvocellular portions of nucleus isthmi (**Imc** or **Ipc**) plotted as a function of either the log-transformed brain volume minus the volume of the respective nuclei (Imc, **A**; Ipc, **C**) or the log-transformed volume of the optic tectum (**TeO**; Imc, **E**; Ipc, **G**) for all species examined (see table S1). The bar graphs show the relative size of each nuclei relative to the brain (Imc, **B**; Ipc, **D**) or the TeO (Imc, **F**; Ipc, **H**). Values shown in the bar graphs are the means of the residuals derived from the respective regressions show in **A**, **C**, **E** and **G**.

**Figure 6:**
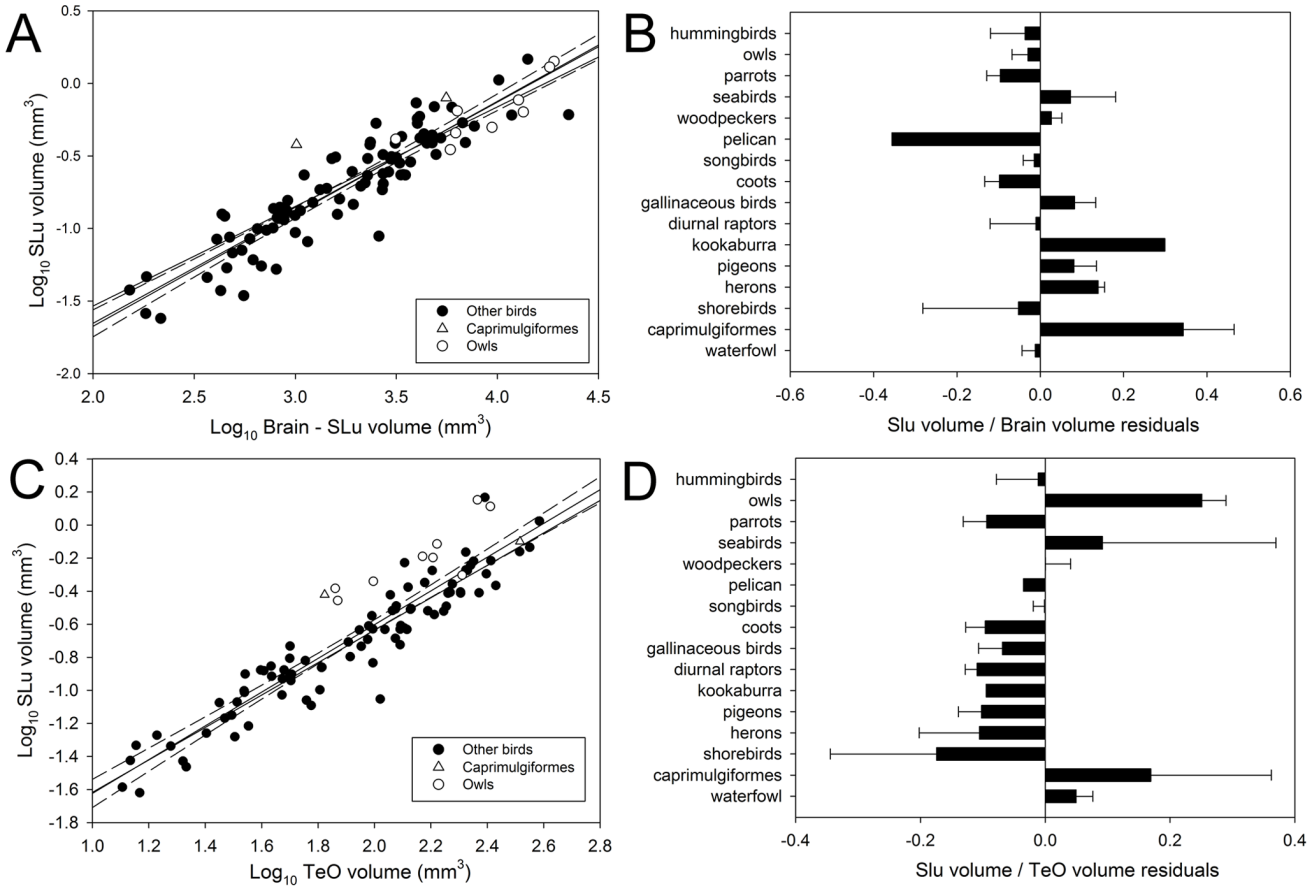
Relative size of nucleus semilunaris. Scatterplot of log-transformed volume of nucleus semilunaris (**SLu**) plotted as a function of the log-transformed brain volume minus the SLu volume (**A**) or the log-transformed volume of the optic tectum (**TeO**; **B**) for all species examined (see table S1). The bar graph shows the relative size of SLu relative to the brain (**B**) or the TeO (**C**). Values shown are the means of the residuals derived from the respective regressions shown in **A** and **C**.

The three isthmal nuclei (Imc, Ipc and SLu) scale with negative allometry against brain volume (table S2; Fig. 5A, C; Fig. 6A). When order is included as a covariate, we found a significant effect of order on the relative size of Imc and Ipc, but not SLu (table S3). Pairwise comparisons using Tukey's HSD test showed that herons, pigeons and gallinaceous birds (i.e., quail, pheasant and relatives) have significantly larger Imc and Ipc volumes than parrots and owls (Fig. 5B, D), relative to brain size. We also tested if species with two layers in Imc (see above, [56]) have relatively larger isthmal nuclei than species with one layer. Species were scored as having a one or two layered Imc, which resulted in two groups: songbirds, Gruiforms and Piciforms (two layers) and all other species (one layer). No significant differences in the relative size of Imc were found between the two groups (table S3).

We also examined the size of the isthmal nuclei relative to the size of the TeO. Imc and Ipc scaled with isometry or positive allometry with the TeO, while SLu scaled with isometry with TeO (Fig. 5E, G; Fig. 6C). This means that as the absolute volume of TeO increases, the size of Imc, Ipc and SLu do so proportionally or slightly more than TeO. When order is included as a covariate, we found a significant effect of orders on the three isthmal nuclei. In the case of Imc and Ipc, songbirds and coots have significantly larger nuclei with respect to the TeO than parrots and hummingbirds (Fig. 5F, H). SLu, however, is larger relative to TeO in owls than most other orders (Fig. 6D).

### Variation in the relative size of other visual nuclei

**Figure 7:**
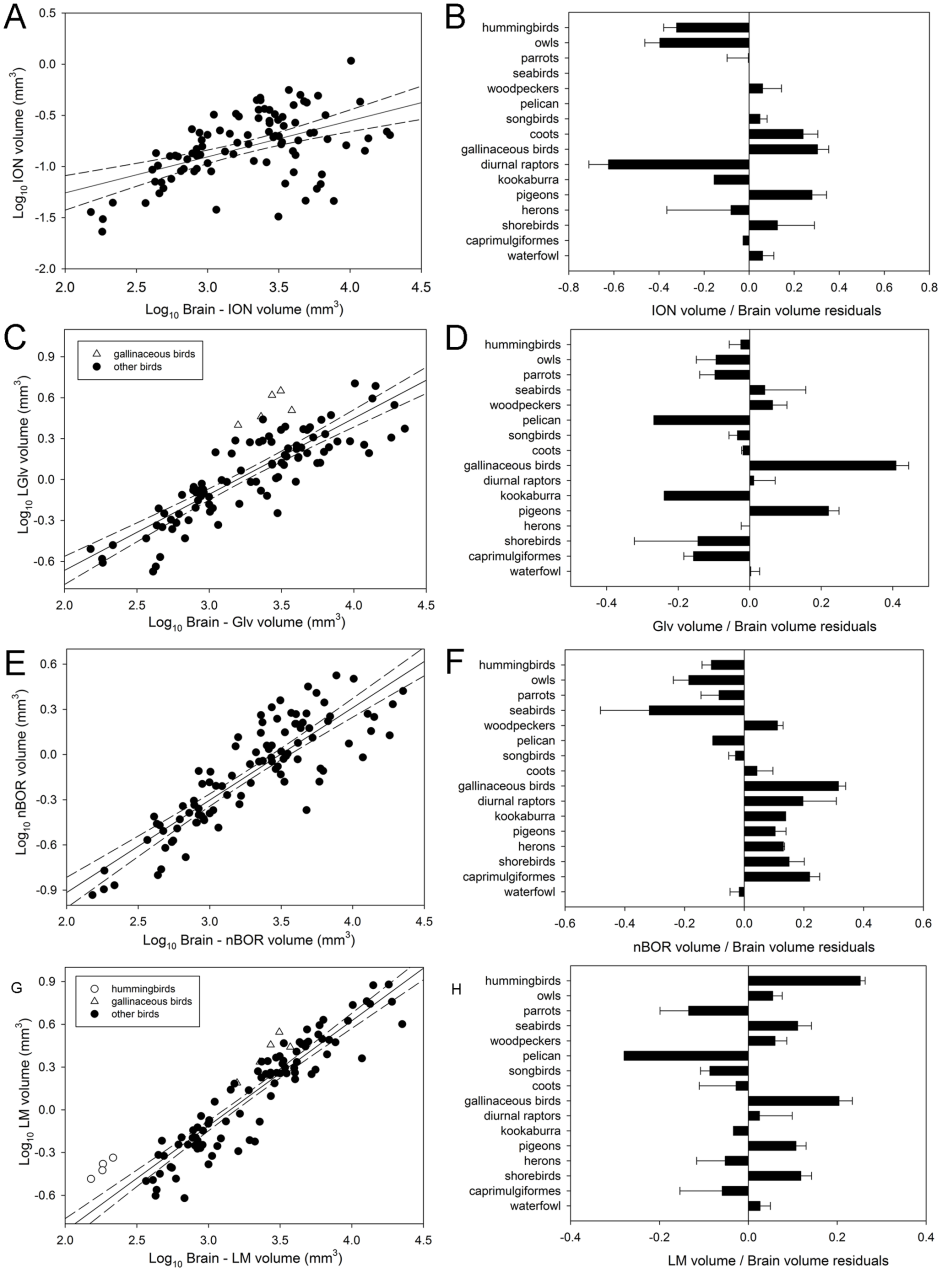
Relative size of other visual nuclei. Scatterplot of log-transformed volume of different nuclei plotted as a function of the log-transformed brain volume minus the volume of the respective nuclei (**A**, **C**, **E**, **G** and **I**). The bar graphs show the relative size each nucleus relative to the brain, represented as the mean of the residuals derived from the respective regressions (**B**, **D**, **F**, **H** and **K**). **A–B**, Scatterplot and bar graph for the isthmo optic nucleus (**ION**). **C–D**, Scatterplot and bar graph for the ventral geniculate nucleus (**GLv**). The white triangles indicate gallinaceous birds and black circles to all other birds studied. **E–F**, Scatterplot and bar graph for the nucleus of the basal optic root (**nBOR**). **G–H**, Scatterplot and bar graph for the nucleus lentiformis mesencephali (**LM**). The white triangles indicate gallinaceous birds, the open circles indicate hummingbirds and the black circles are all other birds species studied.

**Figure 8:**
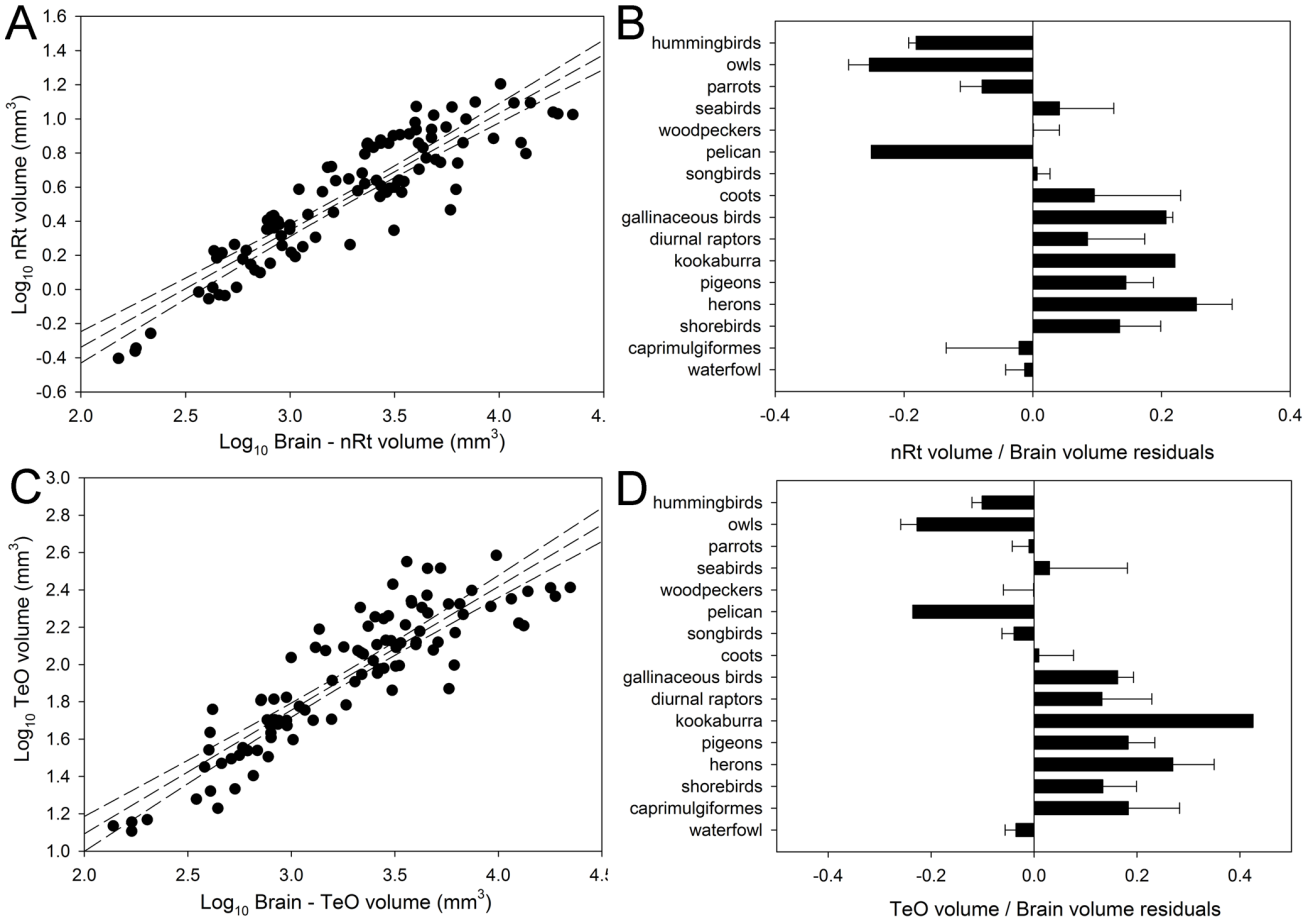
Relative size of optic tectum and nucleus rotundus. Scatterplot of log-transformed volume of structures of the tectofugal pathway plotted as a function of the log-transformed brain volume minus the volume of the respective nuclei (**A** and **C**). The bar graphs show the relative size each nuclei relative to the brain represented as the mean of the residuals derived from the respective regressions (**B** and **D**). **A–B**, Scatterplot and bar graph for the the nucleus rotundus (**nRt**). **C–D**. Scatterplot and bar graph for the optic tectum (**TeO**).

Order also had a significant effect on the relative size of all of the other visual nuclei. Differences in the relative size of ION among orders were not different from those previously reported (Fig. 7A, B; see [55]). GLv and nBOR are significantly larger in gallinaceous birds than most other orders (Fig. 7C–F). Pairwise comparisons show that in the case of LM, hummingbirds and gallinaceous birds have significantly larger LM than parrots, songbirds and the pelican, but not other orders (Fig. 7G, H). Nevertheless, when these two groups are tested against all other species grouped together, they both have significantly larger LM (Fig. 7G, H). Results for TeO and nRt are similar to those reported before [15] with owls and waterfowl having a significantly smaller TeO, relative to brain size, than most other orders (Fig. 8A–B). Parrots had a TeO significantly smaller than pigeons, but not other orders, and a nRT significantly smaller than pigeons, herons and gallinaceous birds.

### Multivariate allometry analysis

We first tested whether the evolutionary rate of change of the log_10_-transformed volumes of each visual nucleus departs significantly from a Brownian motion model using maximum likelihood estimates of α and λ. When the absolute size of the visual nuclei was used, none of them differed significantly from a Brownian model of evolutionary change (table S4). We then performed a multivariate PCA with the log_10_-transformed volume of the visual nuclei using the same two phylogenies as in the regressions (see methods). Although there were some minor differences in the loadings between the two phylogenies (see below, table 1, S5), the overall pattern was similar. The first component of the PCA explained around 80% of the total variance in volume of the different visual nuclei (table 1, S5). All structures loaded strongly and in the same direction in PC1, and species scores for PC1 were significantly correlated with brain size (PGLS using Livezey, and Zusi, (2007; [67]) R^2^ = 0.836, F_1,93_ = 470.2, P = >0.0001). This strongly suggests that PC1 describes variance in the different structures' volumes resulting from differences in brain size. In other words, evolutionary changes in brain size explain about 80% of the variance in the absolute size of the visual nuclei. TeO, nRt Imc, Ipc and SLu had the largest loadings in PC1, which indicates a strong correlation between the volumes of these structures and overall brain size. In contrast, the lower loadings of the other visual nuclei, particularly GLv and ION, suggest a weaker correlation between the volume of these two nuclei and whole brain size. PC2 explained around 7% total variance (table 1). In PC2 GLv has the strongest loading followed by LM. PC3 accounted for 5% of the total variance and ION had a strong positive loading, while GLv and LM loaded weakly in the same direction (table 1).

**Table 1:** Results of principal component analysis.

Hackett et al., 2008 [68]														
log-volume (BM)	PC1	PC2	PC3	PC4	Resid. (BM)	PC1	PC2	PC3	PC4	Resid. (λ)	PC1	PC2	PC3	PC4
Imc	−0.95	0.20	0.03	−0.06		0.84	0.27	0.03	0.15		−0.86	0.20	−0.04	0.07
Ipc	−0.95	0.21	0.06	−0.02		0.90	0.21	0.08	0.04		−0.91	0.21	−0.09	0.06
SLu	−0.91	0.14	0.04	−0.07		0.72	0.06	0.03	0.04		−0.74	0.18	0.02	0.25
ION	−0.72	−0.15	−0.68	0.02		0.28	−0.18	−0.93	−0.09		−0.31	−0.23	0.91	−0.10
GLv	−0.76	−0.58	0.19	0.07		0.29	−0.83	0.15	−0.13		−0.27	−0.82	−0.15	−0.14
nBOR	−0.88	0.05	0.08	0.44		0.57	−0.04	0.14	−0.77		−0.62	−0.06	−0.19	−0.69
LM	−0.87	−0.30	0.09	−0.22		0.43	−0.73	0.08	0.31		−0.35	−0.76	−0.13	0.34
nRt	−0.96	0.11	0.00	−0.02		0.82	0.12	−0.09	0.05		−0.86	0.04	0.09	−0.03
Tectum	−0.95	0.15	0.07	−0.10		0.87	0.08	0.07	0.17		−0.89	0.08	−0.07	0.13
eigenvalues	7.08	0.59	0.52	0.26		4.13	1.39	0.94	0.78		4.31	1.43	0.93	0.71
% variance	78.68	6.53	5.82	2.93		45.88	15.49	10.47	8.65		47.88	15.86	10.28	7.93

Loadings, eigenvalues and cumulative amount of variation explained by four of the components (PC's) obtained from a PCA analysis using the log-transformed volume or the relative size (residuals, see methods) of nine visual nuclei. Values obtained using Hackett et al., (2008; [68]) phylogeny are shown. Values obtained with two different evolutionary models (Brownian motion and pagel's lambda) are also shown for the relative size PCA. For complete values with both phylogenies used in this study see table S5.

Using the loadings of each nucleus on PC1, we calculated bivariate allometric coefficients (table 2). Bivariate allometric coefficients show that TeO varies isometrically with the isthmal nuclei (Imc = 1.00, Ipc = 1.00, and SLu = 1.05) and nRt (0.99), but TeO has a positive allometric relationship with the other nuclei (table 2). Similarly, nRt varies isometrically with the isthmal nuclei (Imc = 1.01, Ipc = 1.01, and SLu = 1.05), but has a positive allometric relationship with the other visual nuclei (table 2). Bivariate allometric coefficients also indicated that the isthmal nuclei vary isometrically with each other, but with positive allometry with the other visual nuclei (table 2). LM and nBOR also varied with positive allometry with respect to ION and GLv, but close to isometry with each other (0.97). Finally, GLv and ION varied isometrically with each other (0.99).

**Table 2:** Visual nuclei bivariate allometric coefficients.

	Ipc	SLu	ION	GLv	nBOR	LM	nRt	TeO
Imc	1	0.96	0.78	0.8	0.93	1.93	1.01	1
Ipc		0.1	0.77	0.8	0.93	1.93	1.01	1
SLu			0.81	0.84	0.97	1.97	1.05	1.05
ION				1.03	1.2	1.19	1.3	1.29
GLv					1.16	1.16	1.26	1.25
nBOR						1	1.09	1.08
LM							1.09	1.08
nRt								0.99

Coefficients of the bivariate allometric relationship between visual nuclei calculated from the loading of each nucleus in the first principal component of a phylogenetically corrected PCA performed with Hacket et al., (2008; [68]) phylogeny (see Methods for calculations details).

We then performed the same analysis as above, but using the relative size of each nucleus expressed as the phylogenetically corrected residuals against the brain. In this case, the evolutionary rate differed significantly from a Brownian motion model for some of the nuclei (table 3). The relative size of SLu, LM and GLv clearly show a significant departure from Brownian motion as both the α and λ Ln likelihood estimates are significantly different from that of the Brownian motion model (table 3). In the case of nBOR and TeO, only the α Ln likelihood estimates are significantly different from that of the Brownian motion model. The evolutionary rate of change of the relative size of ION, Imc, Ipc and nRt, however, are not significantly different from a Brownian motion model.

**Table 3:** Maximum likelihood estimates of the evolutionary parameters.

Hackett et al. (2008; [68])/residuals	Brownian		Lambda			Alpha	
Brain structure	Ln likelihood	λ	Ln likelihood	p	α	Ln likelihood	p
Imc	35.92	1.00	35.92	1.0	0.05	36.16	0.486
Ipc	31.58	1.00	31.58	1.0	0.14	33.38	0.057
SLu	33.03	0.47	39.12	0.0005	0.61	46.08	>0.0001
ION	14.29	1.00	14.29	1.0	0.07	14.92	0.263
GLv	39.10	0.87	41.77	0.021	0.22	42.32	0.011
nBOR	40.82	0.89	42.70	0.052	0.25	44.33	0.008
LM	53.12	0.71	59.89	0.0002	0.23	57.51	0.003
nRt	64.21	1.00	64.21	1	0.07	64.87	0.250
TeO	53.80	0.92	54.82	0.152	0.20	56.99	0.011

Maximum likelihood estimators for the λ and α for the relative size (see methods) of nine visual nuclei. *P* values for the λ and α parameters were determined from likelihood ratio tests against an unconstrained Brownian motion model. Hackett et al. (2008; [68]) phylogeny was used in this case (see table S4 for values with other phylogeny and values obtained with the log-transformed volume of each nuclei).

Because the relative size of some of the nuclei departs from a Brownian motion evolutionary model, we performed a PCA using both a Brownian motion model and Pagel's λ model of evolutionary change. We found no major differences in the estimated values between the two models with either of the phylogenies used (table 1, S5). When relative size of the nuclei was used in the PCA to remove the effect of absolute brain size, the PC1 explained around 45% of the variance. All of the nuclei were positively loaded on PC1, but not with the same strength. Imc, Ipc, TeO and SLu loaded strongly (loadings>0.7) while LM, GLv and ION had loadings well below 0.5 (table 1, S5). PC1 values were significantly correlated with the size of the brain (PGLS using Hackett et al. 2008; R^2^ = 0.109, F_1,93_ = 11.33, P = 0.001), suggesting that the size correction removed most, but not all, effects of variation in brain size. PC2 explains about 15% of the variance with a strong loading of GLv and LM. Finally, PC3 explained about 10.5% of the variance with a strong loading of ION.

## Discussion

This is the first study to assess variation of the relative size of the isthmal nuclei in birds. In recent years, the isthmotectal system has received increased attention, especially in birds, as a model to study visual spatial attention and competitive stimulus selection [93]–[101]. We found the differences in relative size of Ipc and Imc among orders closely matches that of the TeO (Fig. 7J) and the principal component and evolutionary rate analyses further support that Imc and Ipc evolve in a concerted manner with TeO (table 1, 2, 3; see below). Recently Faunes et al. (2013; [56]) showed that Imc is segregated in two distinct layers in at least three different orders; songbirds, woodpeckers and coots, and that these layers correspond, at least in songbirds, to two types of projecting cells in Imc (see Introduction). Our results show that Imc is not relatively larger in any of these three groups compared to other birds. Therefore, the segregation of neurons within Imc is not related to an increase in relative size of the nucleus. Our results do show that there is a significant difference between songbirds, woodpeckers and coots, and the rest of the species in the size of Imc and Ipc relatively to the TeO (Fig. 5E–H; table S3), but woodpeckers do not have a relatively large Imc and Ipc with respect to TeO (Fig. 5E–H). Therefore, the difference in Imc and Ipc size relative to TeO is not entirely due to this separation of two cell layers in Imc. As Faunes et al. [56] pointed out, the segregation of Imc has evolved independently three times, but the groups that have this segregation share little in their ecology or visually guided behaviors, making it difficult to determine the possible functional consequences of this segregation. Lamination of a structure is thought to enhance the separation of information within a neural pathway [102], but that seems to be only partially true in this case. Imc only receives projections from one type of cell in the TeO [52] and even though the segregated cells project to different targets (TeO vs. Ipc/SLu), both inhibit the surrounding of a locus being activated in the TeO and Ipc/SLu [95], [103]. Experiments comparing differences in the responses of the two types of cells segregated in the Imc may be needed to pinpoint the functional consequences of this segregation.

Our results indicate that evolutionary changes in the size of SLu are distinct from that of the other isthmal nuclei. Although bivariate allometric coefficients and loadings of SLu in PC1 of the relative size PCA suggest that the relative size of SLu varies more closely with Imc, Ipc, TeO and nRt, other lines of evidence suggest that the relative size of SLu is more independent. First, the differences among orders in the relative size of Imc and Ipc closely follow the variation in relative size of TeO and nRt, but relative SLu size did not significantly vary among orders. This suggests that the variation in relative size of SLu is different from that of Imc and Ipc (and TeO/nRt). Second, while the evolutionary rate of the relative size of Imc, Ipc, TeO and nRt do not differ significantly from a Brownian motion model (see below), that of SLu clearly does (table 3). The difference in evolutionary patterns between Ipc and SLu is surprising given the similarities between these two nuclei. Both are cholinergic, have reciprocal topographic projections with the TeO, and also receive an anti-topographic projection from Imc, presumably from collaterals of axons going to Ipc (Fig. 1B; [47]). This suggests that, like Ipc, SLu takes part in a stimulus selection mechanism in the TeO, but with different tectal outputs. Ipc projects mainly to the retinorecipient layers of TeO, whereas SLu projects to deeper layers [47]. Within the TeO, Ipc and SLu terminals make contact with different types of tectal ganglion cells (TGCs), Type I and Type II respectively [47], [104], [105]. Type I and II TGCs then project to different targets within nRt [104], [105]. Alternatively, it has recently been suggested that SLu terminals make contact with TGCs that give rise to descending tectal projections [96], the tectopontine and crossed tectobulbar pathways [106], rather than type II TGCs. In either case, Ipc and SLu seem to contact different population of TeO cells and this difference in connectivity between them suggest they differ slightly in function. Our results show that while both nuclei seem to covary in some degree with TeO, they also differ markedly in their evolutionary patterns. This would support the view that there are functional differences between Ipc and SLu.

Interestingly, while we found no differences in size of SLu relative to the brain among orders (Fig. 6B), owls have a greatly enlarged SLu relative to the size of TeO (Fig. 6C–D). As already mentioned (see above), SLu sends projections to the deep layers of TeO, which are the same layers that in owls receive auditory projections from the external part of the inferior colliculus [107], which then results in an auditory spatial map in register with the visual map of the TeO [108]. Owls have enlarged auditory nuclei compared to other birds [10], [109] and thus the large size of SLu relative to the TeO may be related to the largely bimodal nature of the TeO in owls.

### Other visual nuclei

In both PCAs, the second principal component explained around 15% of the variation and GLv and LM were loaded in the same direction, suggesting they vary in relative size together and therefore may have shared functions. Groups like gallinaceous birds and pigeons, which have relatively large LM and GLv, have likely driven this covariation of LM and GLv sizes. In a previous study, Iwaniuk and Wylie [12] showed, using a smaller sample of species, that hummingbirds and other semi hovering species have a large LM compared to other species. Our results confirm these findings, but also show that gallinaceous birds have enlarged LM compared to other birds. This difference between the two studies is likely related to the species sampling. Iwaniuk and Wylie [12] only had one species of gallinaceous birds while we sampled 5, allowing for statistical comparisons with other groups. As mentioned before, the function of GLv remains unknown, but many functions have been proposed (see Introduction). Interestingly, Gioanni et al. [35] showed that in pigeons, lesions of GLv had a marked effect on the gain of the horizontal, but not the vertical, optokinetic nystagmus, especially in the temporal to nasal direction. nBOR and LM are both involved in generating the optokinetic response [29], [110], [111] and have similar response properties [112]–[114], but cells in LM respond preferentially to motion in the temporal-nasal direction. Our results suggesting some covariation of the relative size of LM and GLv would then support the idea that GLv is involved in regulating the optokinetic response, particularly in the temporal-nasal direction. A possible caveat is that projections from nBOR to LM pass immediately dorsal to GLv [42] and therefore lesions of GLv may also lesion this pathway. Inhibition of nBOR has a profound effect on the spatio-temporal tuning of LM cells [115] and therefore the effect of lesioning GLv upon the optokinetic response may be due to the interruption of the nBOR-LM pathway.

Variation in the relative size of the TeO and nRt among orders were similar to what has been reported before [15]. Owls and waterfowl have the smallest TeO and nRt relative size, while diurnal raptors, herons, pigeons and gallinaceous birds have a relatively large TeO and nRt (Fig. 7I–J). In a previous study, we found that parrots have a TeO relatively smaller than most other orders [15], but in our current study, the TeO of parrots is only significantly smaller than that of pigeons. Again, these differences are probably related to differences in species sampling between the two studies. For example, in Iwaniuk et al. [15], 24 species of parrots were sampled whereas in the present study only 8 species were sampled. Fewer species were sampled in our study because it was not always possible to measure the size of all regions of interest due to the quality of the tissue and staining in some of the specimens. Species sampling can affect the slope and intercept of allometric relationships [1], [116] and therefore affect the residuals of different groups. Nonetheless, our results still suggest that parrots have a relatively small tectofugal pathway compared to other birds [117].

### Statistical analysis

Previous studies that tested for differences between mosaic and concerted models of evolutionary change in the brain did so by examining allometric scaling trends (e.g. [4], [8]). Although allometric approaches reveal some important information on brain structure evolution, they are clearly insufficient to adequately assess covariation among structures, particularly covariation in relative size. The use of a combination of statistical approaches, phenotypic evolutionary rates of changes and phylogenetically corrected PCA (pPCA), provides a robust way to assess covariation of the relative size of neural structures. In our study, the concerted variation of isthmal nuclei and TeO and the more independent variation of other visual nuclei were supported by differences/similarities in evolutionary rates of change, bivariate allometric coefficients and the loadings of each structure in different principal components.

Our study also examined both absolute sizes and phylogenetically corrected relative sizes whereas previous studies have only examined one or the other [6], [118] in their pPCAs. As shown above, both methods provide different information. In the pPCa with absolute volume, PC1 reflects isometric changes in the size of the brain and therefore provides us with the bivariate allometric coefficient, which in turn provides a way to test concerted or mosaic evolution. In the pPCa with the size corrected values, while most PCs are very similar to the other analysis, the PC1 revealed a brain size independent covariation of the visual nuclei not shown in the other analysis (see results). Future studies should use a combination of these analyses, in addition to changes in evolutionary rate to properly assess the evolution of brain morphology as they provide multiple, independent means of testing the covariation of different neural structures.

### Multivariate allometric analysis

Our results strongly suggest a combination of mosaic and concerted evolution in the relative size of nine nuclei of the visual system of birds. Across the 98 species of birds we examined, the relative size of the isthmal nuclei (particularly Imc and Ipc) and components of the tectofugal pathway (TeO and nRT) vary together, but the relative volumes of ION, nBOR and ION vary independently of one another in more of a mosaic manner. This pattern is supported by several lines of evidence. First, the bivariate allometric coefficients between Imc, Ipc, SLu, TeO and nRt are all close to 1 (table 2), indicating there is an isometric relationship among the isthmal nuclei, and also between the isthmal nuclei and the tectofugal pathway. In contrast, most of the bivariate allometric coefficients calculated between all other nuclei depart from isometry (table 2) and therefore support a mosaic model. Second, in PC1 of the size corrected PCA (table 1), all nuclei have positively loadings, but the loadings for Imc Ipc, TeO and nRt are much higher than the other visual nuclei. The remaining nuclei only have strong loadings for the other PCs. Again, this strongly indicates that the relative sizes of each of these other nuclei vary independently from one another, or at least only in pairs (e.g. LM and GLv). Third, as mentioned above, the differences in the relative size of Ipc, Imc, TeO and nRt are all similar to one another, further suggesting that these nuclei vary in a concerted manner. Finally, the evolutionary rates of change of the different nuclei also support this claim. In concerted evolutionary models, one would expect nuclei that vary in size together to evolve at the same rate. Our results show that changes in relative size of Imc and Ipc, nRt and TeO do not differ significantly from a Brownian motion model, but GLv, LM, nBOR and SLu do (table 3).

The low degree of covariation in the relative sizes of GLv, nBOR and LM from TeO suggested by our results is somewhat surprising given that all three nuclei receive projections from the retina. Iwaniuk et al. (2010; [15]) suggested that the relative size of the tectofugal pathway is correlated with the relative amount of retinal ganglion cells (RGCs) and subsequent studies appear to support this idea [119], [120]. Owls and waterfowl, which have relatively small tectofugal pathways, have relatively fewer RGCs compared to other birds [119], [120], and in owls, the relative size of the tectofugal pathway is correlated with the relative number of RGCs [17]. Our results show that other retinorecipient nuclei do not vary in relative size along with the TeO and this could suggest that the number of RGCs is unlikely to be associated with the sizes of nBOR, GLv or LM. Support for this hypothesis is provided by the pattern of retinal projections to these nuclei; afferents of each nucleus arise from independent populations of RGCs [28], [29], [121], [122]. So it is possible that while the total amount of RGCs or relative size of TeO increases, the amounts of cells that project to these different nuclei remain unchanged or vary independently of total number of RGCs.

Previous studies have suggested that functionally and anatomically related neural structures should vary together [4], [57]. On the one hand, the concerted variation of the size of the isthmal nuclei and TeO seems to support this notion. The isthmal nuclei and TeO are heavily interconnected (see introduction) and the isthmal nuclei all participate in a circuit related to stimulus selection in the TeO [94], [95], [100]. On the other hand, the independent variation of LM and nBOR, which are also heavily interconnected [42], [123] and functionally related [32], [103], [110], [111], seems to reject the concerted model. This contradictory pattern may be at least partially explained by the diversity of connections of the retinorecipient nuclei. The isthmal nuclei are connected to a much smaller number of other brain regions when compared to the retinorecipient nuclei in this study. Imc only receives projections from TeO and projects to TeO, Ipc and SLu, while Ipc and SLu only receive projections from TeO and Imc (reviewed in [124]). So while only a small fraction of cells in TeO project to the isthmal nuclei, cells in the isthmal nuclei only project to either the TeO or other isthmal nuclei, forming a closed network. This is also supported by the close variation of relative size of three components of the tectofugal pathway (TeO, nRt and entopallium), which was previously suggested by Iwaniuk et al. (2010; [15]) and seems largely confirmed by our results showing that TeO and nRt evolve in a concerted manner. nRt receives projection only from TeO, the nucleus subpretectalis [125] and maybe SLu [46] and projects exclusively to the entopallium which only has one other afferent [126], [127]. In contrast, in addition to receiving projections from the retina and each other, LM and nBOR receive projections from the visual Wulst, the TeO and other structures [128], [129]. LM and nBOR also have a diversity of efferent targets that includes the inferior olive, cerebellum, oculomotor regions, pontine nuclei and ventral tegmentum, among other structures [41], [42], [123], [130], and these projections emerge from distinct neuronal populations within nBOR and LM [131], [132]. Similarly, GLv also has several inputs and outputs; besides efferents from the retina and TeO [38], [39], [133], GLv receives projections from the Wulst [134], [135] and projects to the dorsal thalamus [136] and the TeO [39], [44]. Therefore, the covariation of different neural structures may depend not only on the functional connectivity of each nucleus, but also on the “exclusivity” or diversity of the connections between them.

We think our study further emphasizes the need for future research to consider variation of neural pathways as a whole and not isolated neural structures, particularly when the relative size of a neural structures in being correlated with a particular ecology or behavior. Our study shows that a combination of multivariate statistics and rates of evolution constitute a robust method to study patterns of evolutionary change in neural pathways.

## Supporting Information

Table S1 List of the species surveyed, sample sizes and volumes (mm^3^) of the magnocellular and parvocellular portions of nucleus isthmi (Imc, Ipc), the nucleus semilunaris (SLu), the isthmo optic nucleus (ION), the ventral part of the geniculate nucleus (Glv), the nucleus of the basal optic root (nBOR), the nucleus lentiformis mesencephali, the optic tectum (TeO) and the Brain for each species.
(DOC)

Table S2 Results of least-squares linear regression performed on the log-transformed volume the magnocellular and parvocellular portions of nucleus isthmi (Imc, Ipc), the nucleus semilunaris (SLu), the isthmo optic nucleus (ION), the ventral part of the geniculate nucleus (Glv), the nucleus of the basal optic root (nBOR), the nucleus lentiformis mesencephali, the nucleus rotundus (nRt) and the optic tectum (TeO) against the log-transformed brain volume minus the volume of the respective nuclei are provided using both species as independent data points (‘no phylogeny’) and two models of evolutionary change, Brownian motion (PGLS) and Ornstein-Uhlenbeck (OU) with two different phylogenetic trees.
(DOCX)

Table S3 Results of least-squares linear regression performed on the log-transformed volumes of the magnocellular and parvocellular portions of nucleus isthmi (Imc, Ipc), the nucleus semilunaris (SLu), the isthmo optic nucleus (ION), the ventral part of the geniculate nucleus (Glv), the nucleus of the basal optic root (nBOR), the nucleus lentiformis mesencephali, the nucleus rotundus (nRt) and the optic tectum (TeO) against the log-transformed brain volume minus the volume of the respective nuclei with the order of each species as a covariate. Results are provided using both species as independent data points (‘no phylogeny’) and two models of evolutionary change, Brownian motion (PGLS) and Ornstein-Uhlenbeck (OU) with two different phylogenetic trees. Values for regression of the log-transformed volume of Imc, Ipc and Slu against the log-transformed TeO volume are also provided.
(DOCX)

Table S4 Maximum likelihood estimates of the evolutionary parameters. Maximum likelihood estimators for the λ and α for the the log-transformed volume and the relative size (residuals, see methods) of eight visual nuclei using two different phylogenies. *P* values for the λ and α parameters were determined from likelihood ratio tests against an unconstrained Brownian motion model. Values for the relative size using Livezey and Zusi (2007; [67]) phylogeny are shown in table 1.
(DOCX)

Table S5 Loadings, eigenvalues and cumulative amount of variation explained by four of the components (PC's) obtained from a PCA analysis using the log-transformed volume or the relative size (residuals, see methods) of nine visual nuclei. Values obtained using Livezey and Zusi (2007; [67]) phylogeny are shown.
(DOCX)
