# Supplementary material for: Mosaic and Concerted Evolution in the Visual System of Birds
Source: PLoS One. 2014 Mar 12;9(3):e90102. doi: 10.1371/journal.pone.0090102 (PMC3951201; doi:10.1371/journal.pone.0090102)
Supplement: Table S1 — List of the species surveyed, sample sizes and volumes (mm3) of the magnocellular and parvocellular portions of nucleus isthmi (Imc, Ipc), the nucleus semilunaris (SLu), the isthmo optic nucleus (ION), the ventral part of the geniculate nucleus (Glv), the nucleus of the basal optic root (nBOR), the nucleus lentiformis mesencephali, the optic tectum (TeO) and the Brain for each species. (DOC) [file pone.0090102.s001.doc]

**Table S1**. List of the species surveyed, sample sizes and volumes (mm^3^) of the magnocellular and parvocellular portions of nucleus isthmi (Imc, Ipc), the nucleus semilunaris (SLu), the isthmo optic nucleus (ION), the ventral part of the geniculate nucleus (Glv), the nucleus of the basal optic root (nBOR), the nucleus lentiformis mesencephali (LM), the nucleus rutundus (nRt, the optic tectum (TeO) and the Brain for each species.

| Order | Common name | Species | n | Imc | Ipc | Slu | ION | Glv | nBOR | LM | nRt | Tectum | Brain |
| --- | --- | --- | --- | --- | --- | --- | --- | --- | --- | --- | --- | --- | --- |
| Anseriformes | Green-winged Teal | Anas carolinensis | 1 | 2.122 | 2.481 | 0.309 | 0.198 | 1.327 | 1.048 | 1.804 | 3.97 | 134.19 | 3165.83 |
|  | Chestnut Teal | Anas castanea | 1 | 2.126 | 2.132 | 0.235 | 0.249 | 1.472 | 0.977 | 1.980 | 3.71 | 98.74 | 3424.71 |
|  | Northern Shoveller | Anas clypeata | 1 | 2.719 | 3.098 | 0.283 | 0.165 | 1.279 | 0.935 | 2.104 | 4.32 | 97.93 | 3288.51 |
|  | Blue-winged Teal | Anas discors | 1 | 1.585 | 2.334 | 0.245 | 0.193 | 1.020 | 0.801 | 1.534 | 3.72 | 95.47 | 2895.75 |
|  | Mallard duck | Anas plathyrinchus | 1 | 5.054 | 5.070 | 0.391 | 0.185 | 2.964 | 1.792 | 3.095 | 9.95 | 185.44 | 6949.81 |
|  | Pacific Black Duck | Anas supercilliosa | 1 | 2.691 | 2.969 | 0.324 | 0.420 | 2.413 | 1.495 | 3.011 | 5.77 | 119.49 | 4973.94 |
|  | Lesser Scaup | Athya affinis | 1 | 3.369 | 4.141 | 0.420 | 0.129 | 1.461 | 1.193 | 2.164 | 5.06 | 131.66 | 4141.89 |
|  | Redhead | Athya americana | 1 | 3.488 | 3.797 | 0.421 | 0.212 | 2.035 | 1.292 | 1.779 | 5.55 | 131.70 | 5245.17 |
|  | Bufflehead | Bucephala albeola | 1 | 2.564 | 3.692 | 0.592 | 0.268 | 1.439 | 0.929 | 2.558 | 7.20 | 127.91 | 4122.97 |
|  | Common Goldeneye | Bucephala clangula | 1 | 2.975 | 4.427 | 0.685 | 0.492 | 2.740 | 0.807 | 3.927 | 11.72 | 210.87 | 5961.39 |
|  | Australian Wood Duck | Chemonneta jubata | 1 | 3.205 | 3.550 | 0.449 | 0.180 | 1.716 | 1.498 | 2.985 | 6.77 | 150.79 | 4329.15 |
|  | Red-breasted Merganser | Mergus serrator | 1 | 3.909 | 4.034 | 0.440 | 0.434 | 2.309 | 1.872 | 2.782 | 7.76 | 188.89 | 4754.34 |
| Caprimulgiformes | Spotted Nightjar | Eurostopodus argus | 1 | 5.381 | 8.742 | 0.795 | 0.214 | 1.320 | 2.561 | 1.914 | 1.65 | 66.54 | 5585.91 |
|  | Tawny Frogmouth | Podargus strigoides | 1 | 1.337 | 1.361 | 0.379 |  | 0.580 | 0.769 | 0.845 | 8.95 | 328.05 | 1013.00 |
| Charadriiformes | Silver Gull | Chroicocephalus novaehollandiae | 1 | 3.393 | 3.941 | 0.301 | 0.324 | 0.567 | 1.727 | 2.323 | 7.19 | 176.08 | 2968.15 |
|  | Bonaparte's Gull | Chroicocephalus philadelphia | 1 | 3.511 | 5.843 | 0.531 | 0.365 | 0.759 | 1.146 | 1.785 | 6.80 | 160.31 | 2512.55 |
|  | Eurasian Woodcock | Scolopax rusticola | 1 | 1.178 | 2.041 | 0.088 | 0.110 | 2.072 | 1.086 | 2.198 | 4.36 | 104.92 | 2593.63 |
| Ciconiiformes | Cattle Egret | Bubulcus ibis | 1 | 6.058 | 7.070 | 0.531 | 0.088 | 1.775 | 1.598 | 1.640 | 11.80 | 213.76 | 4025.10 |
|  | Nankeen Night Heron | Nycticorax caledonicus | 1 | 4.968 | 4.796 | 0.431 | 0.305 | 1.439 | 1.404 | 1.932 | 8.07 | 269.32 | 3360.04 |
| Columbiformes | White-headed Pigeon | Columba leucomela | 1 | 3.071 | 3.388 | 0.393 | 0.444 | 2.755 | 0.908 | 2.178 | 7.19 | 201.90 | 2355.21 |
|  | Rock Pigeon | Columba livia | 1 | 3.813 | 5.325 | 0.378 | 0.470 | 1.925 | 1.636 | 1.685 | 7.08 | 113.89 | 2343.44 |
|  | Torresian Imperial Pigeon | Ducula spilorrhoa | 1 | 2.295 | 1.832 | 0.185 | 0.218 | 1.886 | 0.955 | 1.753 | 0.00 | 89.79 | 2697.88 |
|  | Bar-shouldered Dove | Geopelia humeralis | 1 | 2.208 | 2.806 | 0.234 | 0.321 | 1.583 | 0.620 | 1.140 | 3.86 | 108.92 | 1106.18 |
|  | Peaceful Dove | Geopelia placida | 1 | 1.251 | 1.662 | 0.101 | 0.232 | 0.835 | 0.493 | 0.635 | 2.25 | 64.00 | 776.06 |
|  | Wonga Pigeon | Leucosarcia melanoleuca | 1 | 3.175 | 3.022 | 0.206 | 0.445 | 1.880 | 0.897 | 1.865 | 4.81 | 118.75 | 2217.00 |
|  | Brush Bronzewing | Phaps elegans | 1 | 3.834 | 4.367 | 0.303 | 0.132 | 1.931 | 1.133 | 1.528 | 5.19 | 154.58 | 1517.37 |
|  | Spotted Dove | Streptopelia chinensis | 1 | 2.393 | 3.448 | 0.189 | 0.209 | 1.550 | 0.724 | 1.383 | 3.74 | 123.37 | 1430.50 |
| Coraciiformes | Laughing Kookaburra | Dacelo novaeguineae | 1 | 8.131 | 15.165 | 0.733 | 0.141 | 0.962 | 1.598 | 1.964 | 9.52 | 355.42 | 3970.08 |
| Falconiformes | Collared Sparrowhawk | Accipiter cirrocephalus | 1 | 5.547 | 7.218 | 0.690 | 0.046 | 2.361 | 2.824 | 3.661 | 10.50 | 327.20 | 4875.48 |
|  | Swainson's Hawk | Buteo swainsoni | 1 | 7.116 | 5.914 | 0.507 | 0.046 | 1.899 | 3.348 | 2.978 | 12.53 | 249.25 | 7694.02 |
|  | Merlin | Falco columbarius | 1 | 3.542 | 4.045 | 0.234 | 0.068 | 1.691 | 1.014 | 1.803 | 4.29 | 130.46 | 3509.65 |
| Galliformes | Chukar Partridge | Alectoris chukar | 1 | 2.928 | 2.742 | 0.304 | 0.358 | 2.893 | 1.827 | 2.166 | 6.22 | 115.86 | 2284.75 |
|  | Ruffed Grouse | Bonasa umbellus | 1 | 3.959 | 4.721 | 0.387 | 0.285 | 4.470 | 2.287 | 3.512 | 7.97 | 182.33 | 3124.90 |
|  | Spruce Grouse | Falcipennis canadensis | 1 | 2.860 | 4.016 | 0.323 | 0.279 | 4.151 | 2.059 | 2.854 | 7.19 | 179.87 | 2720.00 |
|  | Grey Partridge | Perdix perdix | 1 | 2.536 | 3.370 | 0.310 | 0.327 | 2.504 | 1.303 | 1.547 | 5.25 | 118.69 | 1582.20 |
|  | Common Pheasant | Phasianus colchicus | 1 | 4.032 | 4.455 | 0.287 | 0.559 | 3.210 | 1.888 | 2.758 | 8.15 | 163.03 | 3721.70 |
| Gruiformes | American Coot | Fulica americana | 1 | 3.159 | 3.090 | 0.239 | 0.357 | 1.307 | 1.148 | 1.824 | 7.50 | 127.65 | 2718.92 |
|  | Dusky Moorhen | Gallinula tenebrosa | 1 | 2.365 | 2.064 | 0.203 | 0.265 | 1.285 | 0.898 | 1.249 | 4.05 | 94.62 | 2726.54 |
| Passeriformes | Brown Thornbill | Acanthiza pusilla | 1 | 1.080 | 1.022 | 0.125 | 0.135 | 0.461 | 0.158 | 0.274 | 1.68 | 34.81 | 434.36 |
|  | Eastern Spinebill | Acanthorhynchus tenuirostris | 1 | 0.392 | 0.465 | 0.068 | 0.061 | 0.564 | 0.240 | 0.475 | 0.92 | 29.46 | 489.38 |
|  | Red-winged Blackbird | Agelaius phoeniceus* | 1 | 1.198 | 1.083 | 0.125 | 0.171 | 0.663 | 0.467 | 0.512 | 2.83 | 50.83 | 1614.86 |
|  | Tufted Titmouse | Baeolophus bicolor* | 1 | 1.017 | 0.993 | 0.117 | 0.148 | 0.701 | 0.437 | 0.533 | 2.71 | 47.15 | 837.26 |
|  | Cedar Waxwing | Bombycilla cedrorum | 1 | 0.717 | 0.855 | 0.052 | 0.089 | 0.620 | 0.352 | 0.561 | 1.42 | 31.98 | 805.30 |
|  | American Goldfinch | Carduelis tristis* | 1 | 0.386 | 0.384 | 0.034 | 0.076 | 0.433 | 0.268 | 0.391 | 1.03 | 21.52 | 555.89 |
|  | White-throated Treecreeper | Cormobates leucophaea | 1 | 1.017 | 1.216 | 0.137 | 0.134 | 0.881 | 0.461 | 0.718 | 2.55 | 64.87 | 781.85 |
|  | Australian Magpie | Cracticus tibicen | 1 | 1.221 | 1.470 | 0.185 | 0.140 | 0.959 | 0.538 | 0.828 | 8.63 | 219.43 | 4017.37 |
|  | Gray Catbird | Dumetella carolinensis* | 1 | 0.333 | 0.435 | 0.046 | 0.044 | 0.370 | 0.270 | 0.316 | 2.02 | 50.18 | 1324.32 |
|  | Painted Firetail | Emblema pictum | 1 | 1.909 | 2.839 | 0.131 | 0.127 | 0.831 | 0.777 | 0.752 | 0.97 | 18.96 | 366.80 |
|  | Eastern Yellow Robin | Eopsaltria australis | 1 | 0.384 | 0.572 | 0.037 | 0.071 | 0.230 | 0.346 | 0.249 | 2.70 | 40.52 | 838.80 |
|  | Gouldian Finch | Erythrura gouldiae | 1 | 2.104 | 2.449 | 0.160 | 0.308 | 1.164 | 0.531 | 0.937 | 1.03 | 20.94 | 427.61 |
|  | Rusty Blackbird | Euphagus carolinus | 1 | 5.440 | 5.923 | 0.571 | 0.398 | 1.673 | 1.853 | 1.823 | 4.34 | 82.10 | 1656.56 |
|  | House Finch | Haemorhous mexicanus* | 1 | 0.651 | 0.791 | 0.133 | 0.089 | 0.616 | 0.427 | 0.474 | 1.56 | 39.49 | 1058.98 |
|  | Dark-eyed Junco | Junco hyemalis* | 2 | 1.060 | 1.030 | 0.114 | 0.214 | 0.753 | 0.392 | 0.539 | 2.51 | 50.54 | 879.05 |
|  | White-plumed Honeyeater | Lichenostomus penicillatus | 1 | 0.952 | 0.968 | 0.156 | 0.157 | 0.805 | 0.366 | 0.713 | 1.81 | 50.08 | 916.99 |
|  | Noisy Miner | Manorina melanocephala | 1 | 2.075 | 2.275 | 0.232 | 0.296 | 0.826 | 1.392 | 0.824 | 4.17 | 88.50 | 2278.96 |
|  | Song Sparrow | Melospiza melodia | 1 | 1.077 | 1.233 | 0.133 | 0.181 | 0.846 | 0.375 | 0.567 | 2.06 | 47.79 | 908.78 |
|  | Superb Lyrebird | Menura novaehollandiae | 1 | 13.848 | 14.742 | 1.055 | 1.077 | 5.058 | 3.182 | 5.441 | 16.01 | 384.66 | 10163.13 |
|  | Spotted Pardalote | Pardalotus punctatus | 1 | 1.527 | 1.701 | 0.121 | 0.102 | 0.613 | 0.339 | 0.482 | 1.53 | 43.19 | 447.88 |
|  | Indigo Bunting | Passerina cyanea* | 1 | 0.462 | 0.619 | 0.061 | 0.125 | 0.558 | 0.371 | 0.569 | 1.69 | 35.76 | 618.53 |
|  | Pacific Robin | Petroica multicolor | 1 | 1.113 | 1.704 | 0.087 | 0.070 | 0.448 | 0.311 | 0.606 | 1.64 | 57.40 | 473.94 |
|  | Black-capped Chickadee | Poecile atricapillus | 1 | 1.264 | 1.393 | 0.119 | 0.134 | 0.801 | 0.353 | 0.638 | 2.67 | 50.44 | 814.48 |
|  | Carolina Chickadee | Poecile carolinensis* | 1 | 0.599 | 0.558 | 0.055 | 0.095 | 0.370 | 0.208 | 0.240 | 1.30 | 25.35 | 680.02 |
|  | White-breasted nuthatch | Sitta carolinensis* | 1 | 0.827 | 0.938 | 0.094 | 0.108 | 0.657 | 0.406 | 0.414 | 2.38 | 47.01 | 1000.00 |
|  | Chipping Sparrow | Spizella passerine* | 2 | 0.654 | 0.718 | 0.085 | 0.129 | 0.485 | 0.326 | 0.336 | 1.50 | 32.59 | 595.99 |
|  | Field Sparrow | Spizella pusilla* | 1 | 0.572 | 0.635 | 0.071 | 0.126 | 0.508 | 0.262 | 0.396 | 1.83 | 31.20 | 544.11 |
|  | Diamond Firetail | Stagonopleura guttata | 1 | 0.592 | 0.760 | 0.097 | 0.117 | 0.503 | 0.409 | 0.569 | 1.25 | 34.59 | 720.08 |
|  | Double-barred Finch | Taeniopygia bichenovii | 1 | 0.536 | 0.672 | 0.084 | 0.093 | 0.211 | 0.387 | 0.321 | 0.88 | 28.19 | 409.27 |
|  | Zebra Finch | Taeniopygia guttata | 1 | 0.367 | 0.490 | 0.053 | 0.055 | 0.270 | 0.173 | 0.355 | 0.93 | 16.94 | 458.34 |
|  | Common Blackbird | Tordus merula | 1 | 2.331 | 2.816 | 0.247 | 0.203 | 1.874 | 0.863 | 1.372 | 4.44 | 124.05 | 1914.09 |
|  | House Wren | Troglodytes aedon* | 1 | 0.880 | 0.863 | 0.140 | 0.095 | 0.801 | 0.397 | 0.608 | 2.29 | 42.98 | 839.09 |
|  | White-throated Sparrow | Zonotrichia albicollis* | 1 | 1.370 | 1.344 | 0.151 | 0.225 | 0.989 | 0.617 | 0.629 | 2.75 | 56.88 | 1220.37 |
| Pelecaniformes | Australian Pelican | Pelecanus conspicillatus | 1 | 6.381 | 7.365 | 0.608 |  | 2.358 | 2.640 | 3.995 | 10.58 | 258.77 | 22500.00 |
| Piciformes | Lesser Honeyguide | Indicator minor | 1 | 0.377 | 0.636 | 0.099 | 0.090 | 0.771 | 0.454 | 0.639 | 1.40 | 34.51 | 649.61 |
|  | Downy Woodpecker | Picoides pubescens | 1 | 0.772 | 1.262 | 0.123 | 0.204 | 0.745 | 0.654 | 0.798 | 2.25 | 50.09 | 997.53 |
|  | Yellow-bellied Sapsucker | Sphyrapicus varius | 1 | 0.957 | 1.381 | 0.138 | 0.130 | 0.931 | 0.638 | 0.903 | 2.40 | 65.10 | 888.40 |
| Procellariiformes | Black-browed Albatross | Thalassarche melanophrys | 1 | 6.388 | 6.424 | 1.468 |  | 4.842 | 1.774 | 7.486 | 12.40 | 246.40 | 14129.34 |
|  | Short-tailed Shearwater | Puffinus tenuirostris | 1 | 2.517 | 2.246 | 0.389 |  | 1.561 | 0.428 | 2.915 | 8.67 | 235.01 | 4757.72 |
| Psittasiformes | Australian King Parrot | Alisterus scapularis | 1 | 2.560 | 4.134 | 0.387 | 0.501 | 2.338 | 1.629 | 2.892 | 5.90 | 202.14 | 4478.76 |
|  | Long-billed Corella | Cacatua tenuirostris | 1 | 4.798 | 5.319 | 0.604 | 0.430 | 1.796 | 0.957 | 2.297 | 12.39 | 224.85 | 11777.99 |
|  | Galah | Eolophus roseicapilla | 1 | 3.738 | 4.874 | 0.535 | 0.317 | 1.722 | 1.664 | 2.450 | 7.24 | 211.06 | 6723.94 |
|  | Purple-crowned Lorikeet | Glossopsitta porphyrocephala | 1 | 0.513 | 1.104 | 0.147 | 0.165 | 0.957 | 0.648 | 0.612 | 1.83 | 60.62 | 1939.19 |
|  | Budgerigar | Melopsittacus undulatus | 1 | 0.666 | 0.828 | 0.081 | 0.038 | 0.464 | 0.327 | 0.556 | 1.78 | 59.64 | 1151.54 |
|  | Cockatiel | Nymphicus hollandicus | 1 | 0.866 | 1.412 | 0.196 | 0.113 | 0.962 | 1.034 | 0.598 | 3.79 | 80.82 | 2111.00 |
|  | Superb Parrot | Polytelis swainsonii | 1 | 1.960 | 1.782 | 0.312 | 0.199 | 1.041 | 0.834 | 1.818 | 3.92 | 134.88 | 2996.14 |
|  | Rainbow Lorikeet | Trichoglossus haematodus | 1 | 1.199 | 1.886 | 0.234 | 0.173 | 1.511 | 0.660 | 2.211 | 4.37 | 123.42 | 3333.98 |
| Strigiformes | Northern Saw-whet Owl | Aegolius acadicus | 1 | 1.094 | 1.684 | 0.413 | 0.032 | 2.313 | 0.737 | 2.379 | 2.22 | 72.59 | 3142.86 |
|  | Short-eared Owl | Asio flammeus | 1 | 1.768 | 2.623 | 0.456 | 0.067 | 1.593 | 0.779 | 3.138 | 3.86 | 99.16 | 6221.04 |
|  | Snowy Owl | Bubo scandiacus | 1 | 4.209 | 7.459 | 1.295 | 0.219 | 2.029 | 1.341 | 7.573 | 10.93 | 257.72 | 18127.41 |
|  | Great Horned Owl | Bubo virginianus | 1 | 4.968 | 6.984 | 1.419 | 0.203 | 3.515 | 2.156 | 5.733 | 10.69 | 231.98 | 19073.36 |
|  | Southern Boobook | Ninok boobook | 1 | 3.342 | 4.632 | 0.646 | 0.084 | 2.153 | 2.214 | 4.281 | 5.50 | 148.15 | 6338.80 |
|  | Geat Grey Owl | Strix nebulosa | 1 | 4.197 | 5.660 | 0.636 | 0.188 | 3.921 | 1.431 | 5.537 | 6.25 | 161.30 | 13433.40 |
|  | Barred Owl | Strix varia | 1 | 3.360 | 4.381 | 0.769 | 0.142 | 1.562 | 1.862 | 5.783 | 7.25 | 166.64 | 12727.12 |
|  | Northern Hawk-Owl | Surnia ulula | 1 | 7.118 | 5.600 | 0.498 | 0.161 | 1.907 | 1.182 | 4.213 | 7.68 | 204.75 | 9408.30 |
|  | Barn Owl | Tyto alba | 1 | 1.611 | 1.740 | 0.349 | 0.060 | 1.326 | 0.660 | 3.374 | 2.93 | 74.09 | 5849.81 |
| Trochiliformes | Rufous-tailed Hummingbird | Amazilia tzacatl | 1 | 0.171 | 0.233 | 0.026 | 0.023 | 0.262 | 0.127 | 0.376 | 0.43 | 12.77 | 182.19 |
|  | Anna's Hummingbird | Calypte anna | 1 | 0.095 | 0.189 | 0.046 | 0.031 | 0.245 | 0.170 | 0.417 | 0.45 | 14.28 | 183.88 |
|  | Long-tailed Hermit | Phaethornis superciliosus | 1 | 0.134 | 0.191 | 0.024 | 0.044 | 0.330 | 0.135 | 0.461 | 0.55 | 14.72 | 216.15 |
|  | Rufous Hummingbird | Selasphorus rufus | 1 | 0.143 | 0.216 | 0.038 | 0.036 | 0.309 | 0.117 | 0.327 | 0.39 | 13.63 | 151.64 |

* Capture and handling of this specimens was approved by the Purdue Animal Care and Use Committee (protocol #1201000567), see methods for details.
