# Supplementary material for: Mosaic and Concerted Evolution in the Visual System of Birds
Source: PLoS One. 2014 Mar 12;9(3):e90102. doi: 10.1371/journal.pone.0090102 (PMC3951201; doi:10.1371/journal.pone.0090102)
Supplement: Table S2 — Results of least-squares linear regression performed on the log-transformed volume the magnocellular and parvocellular portions of nucleus isthmi (Imc, Ipc), the nucleus semilunaris (SLu), the isthmo optic nucleus (ION), the ventral part of the geniculate nucleus (Glv), the nucleus of the basal optic root (nBOR), the nucleus lentiformis mesencephali, the nucleus rotundus (nRt) and the optic tectum (TeO) against the log-transformed brain volume minus the volume of the respective nuclei are provided using both species as independent data points (‘no phylogeny’) and two models of evolutionary change, Brownian motion (PGLS) and Ornstein-Uhlenbeck (OU) with two different phylogenetic trees. (DOCX) [file pone.0090102.s002.docx]

**Table S2**. Results of least-squares linear regression performed on the log-transformed volume the magnocellular and parvocellular portions of nucleus isthmi (Imc, Ipc), the nucleus semilunaris (SLu), the isthmo optic nucleus (ION), the ventral part of the geniculate nucleus (Glv), the nucleus of the basal optic root (nBOR), the nucleus lentiformis mesencephali, the nucleus rotundus (nRt) and the optic tectum (TeO) against the log-transformed brain volume minus the volume of the respective nuclei are provided using both species as independent data points (‘no phylogeny’) and two models of evolutionary change, Brownian motion (PGLS) and Ornstein-Uhlenbeck (OU) with two different phylogenetic trees.

|  |  |  |  | **Imc** |  |  |  |  |  |  | **Glv** |  |  |
| --- | --- | --- | --- | --- | --- | --- | --- | --- | --- | --- | --- | --- | --- |
|  | **model** | **d .f.** | **F** | **slope** | **r^2^** | **AIC** |  | **model** | **d .f.** | **F** | **slope** | **r^2^** | **AIC** |
| No phylogeny |  | 1,96 | 284.08 | 0.765 | 0.747 | -18.72 | No phylogeny |  | 1,96 | 217.54 | 0.567 | 0.694 | -50.98 |
| Livezey and Zusi, 2007 | PGLS | 1,96 | 247.92 | 0.914 | 0.721 | -57.95 | Livezey and Zusi, 2007 | PGLS | 1,96 | 79.94 | 0.512 | 0.454 | -60.71 |
|  | OU | 1,96 | 251.29 | 0.896 | 0.724 | -58.49 |  | OU | 1,96 | 115.05 | 0.538 | 0.545 | -69.18 |
| Hackett et al., 2008 | PGLS | 1,98 | 238.39 | 0.911 | 0.713 | -53.51 | Hackett et al., 2008 | PGLS | 1,96 | 85.66 | 0.522 | 0.472 | -62.18 |
|  | OU | 1,98 | 237.71 | 0.890 | 0.712 | -54.21 |  | OU | 1,96 | 116.29 | 0.542 | 0.548 | -70.16 |
|  |  |  |  | **Ipc** |  |  |  |  |  |  | **nBOR** |  |  |
|  | **model** | **d .f.** | **F** | **slope** | **r^2^** | **AIC** |  | **model** | **d .f.** | **F** | **slope** | **r^2^** | **AIC** |
| No phylogeny |  | 1,96 | 298.88 | 0.747 | 0.757 | -28.30 | No phylogeny |  | 1,96 | 277.01 | 0.614 | 0.743 | -59.34 |
| Livezey and Zusi, 2007 | PGLS | 1,96 | 247.92 | 0.914 | 0.721 | -57.95 | Livezey and Zusi, 2007 | PGLS | 1,96 | 161.54 | 0.697 | 0.627 | -69.38 |
|  | OU | 1,96 | 251.29 | 0.896 | 0.724 | -58.49 |  | OU | 1,96 | 189.17 | 0.664 | 0.663 | -76.70 |
|  |  |  |  |  |  |  |  |  |  |  |  |  |  |
| Hackett et al., 2008 | PGLS | 1,96 | 191.38 | 0.853 | 0.666 | -44.89 | Hackett et al., 2008 | PGLS | 1,96 | 159.87 | 0.690 | 0.625 | -68.69 |
|  | OU | 1,96 | 205.31 | 0.816 | 0.681 | -49.87 |  | OU | 1,96 | 183.04 | 0.656 | 0.656 | -75.45 |
|  |  |  |  | **Slu** |  |  |  |  |  |  | **LM** |  |  |
|  | **model** | **d .f.** | **F** | **slope** | **r^2^** | **AIC** |  | **model** | **d .f.** | **F** | **slope** | **r^2^** | **AIC** |
| No phylogeny |  | 1,96 | 506.08 | 0.762 | 0.841 | -75.85 | No phylogeny |  | 1,98 | 537.69 | 0.741 | 0.849 | -87.26 |
| Livezey and Zusi, 2007 | PGLS | 1,96 | 154.63 | 0.617 | 0.748 | -50.93 | Livezey and Zusi, 2007 | PGLS | 1,96 | 173.86 | 0.618 | 0.644 | -99.93 |
|  | OU | 1,96 | 364.08 | 0.760 | 0.791 | -76.53 |  | OU | 1,96 | 244.27 | 0.659 | 0.718 | -106.76 |
| Hackett et al., 2008 | PGLS | 1,96 | 204.57 | 0.863 | 0.681 | -50.31 | Hackett et al., 2008 | PGLS | 1,96 | 180.86 | 0.626 | 0.653 | -99.75 |
|  | OU | 1,96 | 221.55 | 0.829 | 0.698 | -54.86 |  | OU | 1,96 | 248.13 | 0.663 | 0.721 | -107.26 |
|  |  |  |  | **ION** |  |  |  |  |  |  | **TeO** |  |  |
|  | **model** | **d .f.** | **F** | **slope** | **r^2^** | **AIC** |  | **model** | **d .f.** | **F** | **slope** | **r^2^** | **AIC** |
| No phylogeny |  | 1,96 | 20.74 | 0.313 | 0.178 | 62.51 | No phylogeny |  | 1,96 | 371.01 | 0.663 | 0.794 | -70.18 |
| Livezey and Zusi, 2007 | PGLS | 1,96 | 59.25 | 0.681 | 0.382 | 24.52 | Livezey and Zusi, 2007 | PGLS | 1,96 | 176.58 | 0.663 | 0.648 | -84.78 |
|  | OU | 1,96 | 53.61 | 0.634 | 0.358 | 24.43 |  | OU | 1,96 | 234.84 | 0.669 | 0.710 | -94.38 |
| Hackett et al., 2008 | PGLS | 1,96 | 49.28 | 0.647 | 0.339 | 33.93 | Hackett et al., 2008 | PGLS | 1,96 | 166.41 | 0.652 | 0.634 | -80.81 |
|  | OU | 1,96 | 44.09 | 0.588 | 0.315 | 31.12 |  | OU | 1,96 | 218.69 | 0.658 | 0.695 | -90.14 |
|  |  |  |  | **nRt** |  |  |  |  |  |  |  |  |  |
|  | **model** | **d .f.** | **F** | **slope** | **r^2^** | **AIC** |  |  |  |  |  |  |  |
| No phylogeny |  | 1,96 | 418.27 | 0.686 | 0.813 | -77.73 |  |  |  |  |  |  |  |
| Livezey and Zusi, 2007 | PGLS | 1,96 | 288.34 | 0.759 | 0.750 | -109.95 |  |  |  |  |  |  |  |
|  | OU | 1,96 | 315.77 | 0.746 | 0.766 | -113.67 |  |  |  |  |  |  |  |
| Hackett et al., 2008 | PGLS | 1,96 | 272.65 | 0.745 | 0.739 | -105.73 |  |  |  |  |  |  |  |
|  | OU | 1,96 | 294.06 | 0.735 | 0.753 | -109.51 |  |  |  |  |  |  |  |
|  |  |  |  |  |  |  |  |  |  |  |  |  |  |
