# Supplementary material for: Mosaic and Concerted Evolution in the Visual System of Birds
Source: PLoS One. 2014 Mar 12;9(3):e90102. doi: 10.1371/journal.pone.0090102 (PMC3951201; doi:10.1371/journal.pone.0090102)
Supplement: Table S3 — Results of least-squares linear regression performed on the log-transformed volumes of the magnocellular and parvocellular portions of nucleus isthmi (Imc, Ipc), the nucleus semilunaris (SLu), the isthmo optic nucleus (ION), the ventral part of the geniculate nucleus (Glv), the nucleus of the basal optic root (nBOR), the nucleus lentiformis mesencephali, the nucleus rotundus (nRt) and the optic tectum (TeO) against the log-transformed brain volume minus the volume of the respective nuclei with the order of each species as a covariate. Results are provided using both species as independent data points (‘no phylogeny’) and two models of evolutionary change, Brownian motion (PGLS) and Ornstein-Uhlenbeck (OU) with two different phylogenetic trees. Values for regression of the log-transformed volume of Imc, Ipc and Slu against the log-transformed TeO volume are also provided. (DOCX) [file pone.0090102.s003.docx]

**Table S3**. Results of least-squares linear regression performed on the log-transformed volume the magnocellular and parvocellular portions of nucleus isthmi (Imc, Ipc), the nucleus semilunaris (SLu), the isthmo optic nucleus (ION), the ventral part of the geniculate nucleus (Glv), the nucleus of the basal optic root (nBOR), the nucleus lentiformis mesencephali, the nucleus rotundus (nRt) and the optic tectum (TeO) against the log-transformed brain volume minus the volume of the respective nuclei with the order of each species as a covariate. Results are provided using both species as independent data points (‘no phylogeny’) and two models of evolutionary change, Brownian motion (PGLS) and Ornstein-Uhlenbeck (OU) with two different phylogenetic trees. Values for regression of the log-transformed volume of Imc, Ipc and Slu against the log-transformed TeO volume are also provided.

ff

| Covariates |  | Imc | | | | Ipc | | | | SLu | | | |
| --- | --- | --- | --- | --- | --- | --- | --- | --- | --- | --- | --- | --- | --- |
| Brain / order | Model | d .f. | F | p | AIC | d.f. | F | p | AIC | d.f. | F | p | AIC |
| No phylogeny |  | 15, 81 | 8.58 | < 0 .0001 | -81.95 | 15,81 | 6.83 | < 0 .0001 | 78.39 | 15,81 | 2.21 | < 0.05 | -79.46 |
| Livezey and Zusi, 2007 | PGLS | 15, 81 | 3.16 | < 0.001 | -73.28 | 15,81 | 3.25 | < 0.001 | -66.52 | 15,81 | 1.14 | 0.34 | -39.69 |
|  | OU | 15, 81 | 3.92 | < 0 .0001 | -87.78 | 15,81 | 3.75 | < 0 .0001 | -82.53 | 15,81 | 1.68 | 0.07 | -77.48 |
| Hackett et al., 2008 | PGLS | 15, 81 | 3.48 | < 0.001 | -72.32 | 15,81 | 3.25 | < 0.01 | -65.55 | 15,81 | 1.13 | 0.34 | -39.69 |
|  | OU | 15, 81 | 4.34 | < 0 .0001 | -87.77 | 15,81 | 4.27 | < 0 .0001 | -82.61 | 15,81 | 1.67 | 0.07 | -77.48 |
| TeO / order | Model | d.f. | F | p | AIC | d.f. | F | p | AIC | d.f. | F | p | AIC |
| No phylogeny |  | 15, 81 | 4.24 | < 0 .0001 | -115.39 | 15,81 | 2.86 | < 0.01 | -138.67 | 15,81 | 4.55 | < 0.0001 | -115.68 |
| Livezey and Zusi, 2007 | PGLS | 15, 81 | 0.63 | 0.84 | -64.39 | 15,81 | 0.54 | 0.91 | -94.86 | 15,81 | 0.68 | 0.80 | -53.93 |
|  | OU | 15, 81 | 4.13 | < 0 .0001 | -113.39 | 15,81 | 2.31 | < 0.01 | -136.67 | 15,81 | 4.54 | < 0.0001 | -113.68 |
| Hackett et al., 2008 | PGLS | 15, 81 | 0.66 | 0.81 | -63.73 | 15,81 | 0.58 | 0.88 | -94.69 | 15,81 | 0.70 | 0.78 | -53.10 |
|  | OU | 15, 81 | 4.13 | < 0 .0001 | -113.39 | 15,81 | 2.28 | < 0.01 | -136.66 | 15,81 | 4.54 | < 0.0001 | -113.68 |
| Brain / Imc layers | Model | d .f. | F | p | AIC | d.f. | F | p | AIC | d.f. | F | p | AIC |
| No phylogeny |  | 1,95 | 1.67 | 0.199 | -18.43 | 1,95 | 0.0005 | 0.98 | -26.30 | 1,95 | 0.84 | 0.36 | -74.71 |
| Livezey and Zusi, 2007 | PGLS | 1,95 | 0.46 | 0.501 | -56.42 | 1,95 | 3.36 | 0.07 | -51.72 | 1,95 | 0.50 | 0.48 | -49.44 |
|  | OU | 1,95 | 0.15 | 0.700 | -56.53 | 1,95 | 1.75 | 0.19 | -53.82 | 1,95 | 0.69 | 0.41 | -75.26 |
| Hackett et al., 2008 | PGLS | 1,95 | 0.11 | 0.744 | -51.62 | 1,95 | 0.11 | 0.74 | -43.00 | 1,95 | 0.00 | 0.95 | -45.94 |
|  | OU | 1,95 | 0.30 | 0.587 | -52.64 | 1,95 | 0.01 | 0.92 | -47.87 | 1,95 | 0.58 | 0.45 | -74.14 |
| TeO / Imc layers | Model | d .f. | F | p | AIC | d.f. | F | p | AIC | d.f. | F | p | AIC |
| No phylogeny |  | 1,95 | 15.62 | > 0.001 | -101.46 | 1,95 | 6.49 | 0.01 | -131.42 | 1,95 | 0.14 | 0.71 | -83.98 |
| Livezey and Zusi, 2007 | PGLS | 1,95 | 0.80 | 0.372 | -82.43 | 1,95 | 0.21 | 0.64 | -113.72 | 1,95 | 0.29 | 0.59 | -70.61 |
|  | OU | 1,95 | 8.07 | 0.006 | -103.70 | 1,95 | 3.00 | 0.09 | -133.58 | 1,95 | 0.05 | 0.83 | -89.28 |
| Hackett et al., 2008 | PGLS | 1,95 | 0.43 | 0.515 | -80.85 | 1,95 | 0.009 | 0.92 | -112.63 | 1,95 | 0.03 | 0.86 | -69.17 |
|  | OU | 1,95 | 8.45 | 0.005 | -103.14 | 1,95 | 3.517 | 0.06 | -133.81 | 1,95 | 0.08 | 0.77 | -89.19 |
| Brain / Imc layers | Model | d .f. | F | p | AIC | d.f. | F | p | AIC | d.f. | F | p | AIC |
|  |  | ION | | | | Glv | | | | nBOR | | | |
| Brain / order | Model | d .f. | F | p | AIC | d.f. | F | p | AIC | d.f. | F | p | AIC |
| No phylogeny |  | 15, 81 | 17.52 | < 0 .0001 | -49.17 | 15,81 | 7.14 | < 0 .0001 | -103.56 | 15,81 | 7.20 | < 0 .0001 | -112.37 |
| Livezey and Zusi, 2007 | PGLS | 15, 81 | 4.16 | < 0 .0001 | -1.49 | 15,81 | 1.58 | 0.10 | -55.92 | 15,81 | 1.29 | 0.22 | -60.37 |
|  | OU | 15, 81 | 16.58 | < 0 .0001 | -47.17 | 15,81 | 7.13 | < 0 .0001 | -101.56 | 15,81 | 7.09 | < 0 .0001 | -110.37 |
| Hackett et al., 2008 | PGLS | 15, 81 | 5.27 | < 0 .0001 | -2.79 | 15,81 | 1.45 | 0.14 | -55.43 | 15,81 | 1.43 | 0.15 | -61.75 |
|  | OU | 15, 81 | 16.35 | < 0 .0001 | -47.17 | 15,81 | 7.13 | < 0 .0001 | -101.56 | 15,81 | 7.05 | < 0 .0001 | -110.37 |
|  |  | LM | | | | nRt | | | | TeO | | | |
| Brain / order | Model | d .f. | F | p | AIC | d.f. | F | p | AIC | d.f. | F | p | AIC |
| No phylogeny |  | 15, 81 | 6.67 | < 0 .0001 | -136.06 | 15,81 | 10.60 | < 0 .0001 | -154.19 | 15,81 | 7.72 | < 0 .0001 | -127.21 |
| Livezey and Zusi, 2007 | PGLS | 15, 81 | 1.02 | 0.44 | -86.89 | 15,81 | 2.90 | < 0.01 | -122.13 | 15,81 | 2.52 | < 0.01 | -92.26 |
|  | OU | 15, 81 | 6.67 | < 0 .0001 | -134.06 | 15,81 | 6.05 | < 0 .0001 | -152.75 | 15,81 | 4.47 | < 0 .0001 | -125.21 |
| Hackett et al., 2008 | PGLS | 15, 81 | 1.01 | 0.45 | -86.63 | 15,81 | 3.41 | < 0.001 | -123.69 | 15,81 | 2.88 | < 0.01 | -92.70 |
|  | OU | 15, 81 | 6.67 | < 0 .0001 | -134.06 | 15,81 | 6.29 | < 0 .0001 | -152.87 | 15,81 | 4.94 | < 0 .0001 | -125.21 |
