# Supplementary material for: Mosaic and Concerted Evolution in the Visual System of Birds
Source: PLoS One. 2014 Mar 12;9(3):e90102. doi: 10.1371/journal.pone.0090102 (PMC3951201; doi:10.1371/journal.pone.0090102)
Supplement: Table S4 — Maximum likelihood estimates of the evolutionary parameters. Maximum likelihood estimators for the λ and α for the the log-transformed volume and the relative size (residuals, see methods) of eight visual nuclei using two different phylogenies. P values for the λ and α parameters were determined from likelihood ratio tests against an unconstrained Brownian motion model. Values for the relative size using Livezey and Zusi (2007; [67]) phylogeny are shown in table 1. (DOCX) [file pone.0090102.s004.docx]

**Table S4. Maximum likelihood estimates of the evolutionary parameters.** Maximum likelihood estimators for the λ and α for the log-transformed volume and the relative size (residuals, see methods) of eight visual nuclei using two different phylogenies. *P* values for the λ and α parameters were determined from likelihood ratio tests against an unconstrained Brownian motion model. Values for the relative size using Livezey and Zusi (2007) phylogeny are shown in table 1.

|  |  |  |  |  |  |  |  |
| --- | --- | --- | --- | --- | --- | --- | --- |
| Livezey and Zusi (2007)/log-volume | brownian |  | Lambda |  |  | Alpha |  |
| brain structure | Ln likelihood | lambda | Ln likelihood | p | alpha | Ln likelihood | p |
| Imc | -31.75 | 1.00 | -31.75 | 1.00 | 0.13 | -30.90 | 0.194 |
| Ipc | -28.27 | 1.00 | -28.27 | 1.00 | 0.12 | -27.37 | 0.180 |
| Slu | -19.26 | 0.91 | -18.60 | 0.25 | 0.09 | -18.62 | 0.258 |
| ION | -15.90 | 1.00 | -15.90 | 1.00 | 0.21 | -13.19 | 0.020 |
| Glv | 9.65 | 0.94 | 10.21 | 0.29 | 0.02 | 9.70 | 0.739 |
| nBOR | -9.62 | 0.81 | -8.38 | 0.11 | 0.11 | -9.08 | 0.296 |
| LM | 2.56 | 0.89 | 3.07 | 0.31 | 0.01 | 2.57 | 0.874 |
| Tectum | -8.57 | 1.00 | -8.57 | 1.00 | 0.04 | -8.45 | 0.620 |
|  |  |  |  |  |  |  |  |
| Hackett et al., (2008)/log-volume | brownian |  | Lambda |  |  | Alpha |  |
| brain structure | Ln likelihood | lambda | Ln likelihood | p | alpha | Ln likelihood | p |
| Imc | -32.43 | 1.00 | -32.43 | 1.00 | 0.15 | -30.98 | 0.089 |
| Ipc | -29.03 | 1.00 | -29.03 | 1.00 | 0.15 | -27.49 | 0.080 |
| Slu | -19.63 | 1.00 | -19.62 | 0.92 | 0.11 | -18.60 | 0.153 |
| ION | -17.38 | 1.00 | -17.38 | 0.54 | 0.22 | -14.07 | 0.010 |
| Glv | 9.35 | 0.98 | 9.54 | 0.93 | 0.03 | 9.42 | 0.704 |
| nBOR | -9.73 | 1.00 | -9.72 | 0.52 | 0.14 | -8.67 | 0.145 |
| LM | 1.91 | 0.97 | 2.11 | 0.52 | 0.02 | 1.95 | 0.765 |
| Tectum | -8.99 | 1.00 | -8.99 | 1.00 | 0.09 | -8.43 | 0.287 |
|  |  |  |  |  |  |  |  |
| Hackett et al., (2008)/residuals | brownian |  | Lambda |  |  | Alpha |  |
| brain structure | Ln likelihood | lambda | Ln likelihood | p | alpha | Ln likelihood | p |
| Imc | 33.29 | 1.00 | 33.29 | 1.00 | 0.05 | 33.52 | 0.4990 |
| Ipc | 27.89 | 1.00 | 27.89 | 1.00 | 0.16 | 29.73 | 0.0553 |
| Slu | 30.96 | 0.086 | 36.34 | 0.0010 | 0.81 | 45.40 | > 0.0001 |
| ION | 8.68 | 1.00 | 8.68 | 1.00 | 0.11 | 10.60 | 0.0498 |
| Glv | 39.63 | 0.87 | 42.75 | 0.01 | 0.22 | 42.82 | 0.0115 |
| nBOR | 40.41 | 0.85 | 43.73 | 0.01 | 0.26 | 43.90 | 0.0082 |
| LM | 53.02 | 0.55 | 63.33 | > 0.0001 | 0.24 | 57.66 | 0.0023 |
| Tectum | 51.01 | 0.91 | 51.79 | 0.21 | 0.21 | 54.40 | 0.0092 |
