# Supplementary material for: Mosaic and Concerted Evolution in the Visual System of Birds
Source: PLoS One. 2014 Mar 12;9(3):e90102. doi: 10.1371/journal.pone.0090102 (PMC3951201; doi:10.1371/journal.pone.0090102)
Supplement: Table S5 — Loadings, eigenvalues and cumulative amount of variation explained by four of the components (PC's) obtained from a PCA analysis using the log-transformed volume or the relative size (residuals, see methods) of nine visual nuclei. Values obtained using Livezey and Zusi (2007; [67]) phylogeny are shown. (DOCX) [file pone.0090102.s005.docx]

| Livezey and Zusi (2007) | | | | | | | | | | | | | | |
| --- | --- | --- | --- | --- | --- | --- | --- | --- | --- | --- | --- | --- | --- | --- |
| log-volume  (BM) | PC1 | PC2 | PC3 | PC4 | Resid.  (BM) | PC1 | PC2 | PC3 | PC4 | Resid.  (λ) | PC1 | PC2 | PC3 | PC4 |
| Imc | -0.95 | 0.20 | -0.03 | -0.06 |  | 0.83 | 0.28 | -0.07 | 0.13 |  | -0.86 | 0.23 | 0.06 | 0.05 |
| Ipc | -0.95 | 0.22 | 0.03 | -0.02 |  | 0.90 | 0.23 | 0.04 | 0.03 |  | -0.90 | 0.22 | -0.06 | 0.05 |
| Slu | -0.91 | 0.15 | 0.02 | -0.06 |  | 0.70 | 0.06 | 0.04 | 0.03 |  | -0.72 | 0.16 | 0.01 | 0.24 |
| ION | -0.73 | -0.30 | -0.61 | 0.02 |  | 0.21 | -0.33 | -0.91 | -0.07 |  | -0.27 | -0.35 | 0.89 | -0.10 |
| Glv | -0.76 | -0.54 | 0.29 | 0.06 |  | 0.28 | -0.82 | 0.19 | -0.11 |  | -0.26 | -0.81 | -0.18 | -0.15 |
| nBOR | -0.88 | 0.06 | 0.07 | 0.44 |  | 0.56 | -0.06 | 0.13 | -0.79 |  | -0.61 | -0.10 | -0.22 | -0.67 |
| LM | -0.88 | -0.25 | 0.15 | -0.23 |  | 0.44 | -0.69 | 0.18 | 0.33 |  | -0.37 | -0.71 | -0.19 | 0.41 |
| nRt | -0.96 | 0.11 | -0.03 | -0.02 |  | 0.81 | 0.11 | -0.10 | 0.04 |  | -0.85 | 0.07 | 0.06 | -0.05 |
| TeO | -0.95 | 0.17 | 0.04 | -0.10 |  | 0.87 | 0.09 | 0.08 | 0.17 |  | -0.88 | 0.08 | -0.05 | 0.12 |
| eigenvalues | 7.11 | 0.60 | 0.48 | 0.26 |  | 4.02 | 1.42 | 0.95 | 0.79 |  | 4.21 | 1.43 | 0.92 | 0.73 |
| % variance | 79.02 | 6.71 | 5.37 | 2.93 |  | 44.68 | 15.77 | 10.50 | 8.79 |  | 46.77 | 15.88 | 10.18 | 8.11 |
|  |  |  |  |  |  |  |  |  |  |  |  |  |  |  |

**Table S5**. Loadings, eigenvalues and cumulative amount of variation explained by four of the components (PC’s) obtained from a PCA analysis using the log-transformed volume or the relative size (residuals, see methods) of nine visual nuclei. Values obtained using Livezey and Zusi (2007) phylogeny are shown.
